# Elevating CDCA3 levels in non-small cell lung cancer enhances sensitivity to platinum-based chemotherapy

**DOI:** 10.1038/s42003-021-02136-8

**Published:** 2021-05-28

**Authors:** Katrina Kildey, Neha S. Gandhi, Katherine B. Sahin, Esha T. Shah, Eric Boittier, Pascal H. G. Duijf, Christopher Molloy, Joshua T. Burgess, Sam Beard, Emma Bolderson, Amila Suraweera, Derek J. Richard, Kenneth J. O’Byrne, Mark N. Adams

**Affiliations:** 1 Institute of Health and Biomedical Innovation, School of Biomedical Sciences, Faculty of Health, Queensland University of Technology, Translational Research Institute, Woolloongabba, QLD Australia; 2 Institute of Health and Biomedical Innovation, School of Mathematical Sciences, Faculty of Science and Engineering, Queensland University of Technology, Brisbane, QLD Australia; 3 Cancer Services, Princess Alexandra Hospital, Woolloongabba, QLD Australia

**Keywords:** Non-small-cell lung cancer, Prognostic markers

## Abstract

Platinum-based chemotherapy remains the cornerstone of treatment for most non-small cell lung cancer (NSCLC) cases either as maintenance therapy or in combination with immunotherapy. However, resistance remains a primary issue. Our findings point to the possibility of exploiting levels of cell division cycle associated protein-3 (CDCA3) to improve response of NSCLC tumours to therapy. We demonstrate that in patients and in vitro analyses, CDCA3 levels correlate with measures of genome instability and platinum sensitivity, whereby CDCA3^high^ tumours are sensitive to cisplatin and carboplatin. In NSCLC, CDCA3 protein levels are regulated by the ubiquitin ligase APC/C and cofactor Cdh1. Here, we identified that the degradation of CDCA3 is modulated by activity of casein kinase 2 (CK2) which promotes an interaction between CDCA3 and Cdh1. Supporting this, pharmacological inhibition of CK2 with CX-4945 disrupts CDCA3 degradation, elevating CDCA3 levels and increasing sensitivity to platinum agents. We propose that combining CK2 inhibitors with platinum-based chemotherapy could enhance platinum efficacy in CDCA3^low^ NSCLC tumours and benefit patients.

## Abstract (web summary)

Kildey et al find that high levels of mitotic regulator CDCA3 correlates with sensitivity to platinum agents in non-small cell lung cancer patients and cell lines. They show that interfering with CDCA3 degradation through CK2 inhibition enhances CDCA3 levels and increases sensitivity to platinum agents suggesting a therapeutic route.

## Introduction

Lung cancer is the leading cause of cancer-related mortality accounting for 18.4% of all cancer-related deaths worldwide^1,2^. The most commonly diagnosed form of lung cancer is non-small-cell lung cancer (NSCLC) which is broadly subdivided into the adenocarcinoma (ADC) and squamous cell carcinoma (SqCC) histologies. Patients with NSCLC may undergo adjuvant chemotherapy for resectable disease, chemoradiotherapy, and maintenance immunotherapy for locally advanced disease or systemic treatment (chemotherapy, immunotherapy, and targeted agents) for advanced disease and palliative care. However, despite improvements in these strategies and the introduction of targeted therapies (including immunotherapy), the global 5-year survival rate remains poor at 19%^3^.

Chemotherapy employing platinum-based doublet combinations remains the cornerstone of treatment for non-oncogene driven NSCLC. Cisplatin, *cis*-Diamineplatinum (II) dichloride, is a common platinum agent used in the adjuvant setting, and in combination with radiation or immunotherapy for advanced NSCLC. The mechanism of action for cisplatin is the induction of DNA damage via the formation of inter- or intrastrand crosslinks (ICLs). These crosslinks impede DNA strand separation which occur during the cellular processes of replication fork progression and transcription^4^. While most NSCLC patients receive platinum-based chemotherapy, intrinsic or acquired resistance remains a primary issue with <50% of patients having an objective partial response^5–7^. Identifying and providing alternative treatment options for those patients who will not benefit from platinum-based chemotherapy might improve NSCLC patient health outcomes. Hence, implementing biomarkers or additional combinational therapies is key to improving the effectiveness of platinum-based chemotherapy.

In the search for novel biomarkers or therapeutic targets, we have studied the protein cell division cycle associated protein-3 (CDCA3), also known as trigger of mitosis entry 1 (TOME-1). This protein primarily modulates cell cycle progression from the G2 phase to mitosis. CDCA3 reportedly forms part of a SKP1-Cullin-RING-F-box (SCF) ubiquitin ligase (E3) protein complex to promote the timely degradation of the inhibitory tyrosine kinase WEE1^8,9^. While other molecular functions are yet to be determined, CDCA3 is associated with an emerging role in solid malignancies with upregulated expression noted in oral squamous cell carcinoma tissues^10^, liver cancer^11^, gastric cancer^12,13^, colon cancer^14,15^ and breast cancer^16,17^. We have previously identified a role for CDCA3 in NSCLC^18^. CDCA3 is commonly upregulated in NSCLC versus non-malignant tissue where elevated malignant expression of CDCA3 strongly correlates with poor patient outcomes. We also identified that CDCA3 is functionally important in mediating proficient G2/M cell cycle progression and tumour cell proliferation where depletion of this protein induces tumour cell senescence.

In the present study, we examined the potential of CDCA3 as a biomarker for tumour cell response to platinum-based chemotherapy. Our data demonstrate that CDCA3 levels, in patients and in vitro, correlate strongly with sensitivity to platinum agents. We also demonstrate that while CDCA3 protein levels are regulated by the anaphase-promoting complex/cyclosome (APC/C) in NSCLC, pharmacological strategies to block this protein–protein interaction enhance sensitivity to platinum agents.

## Results

### CDCA3 levels correlate with genome instability and chemotherapy sensitivity in NSCLC

**Fig. 1:**
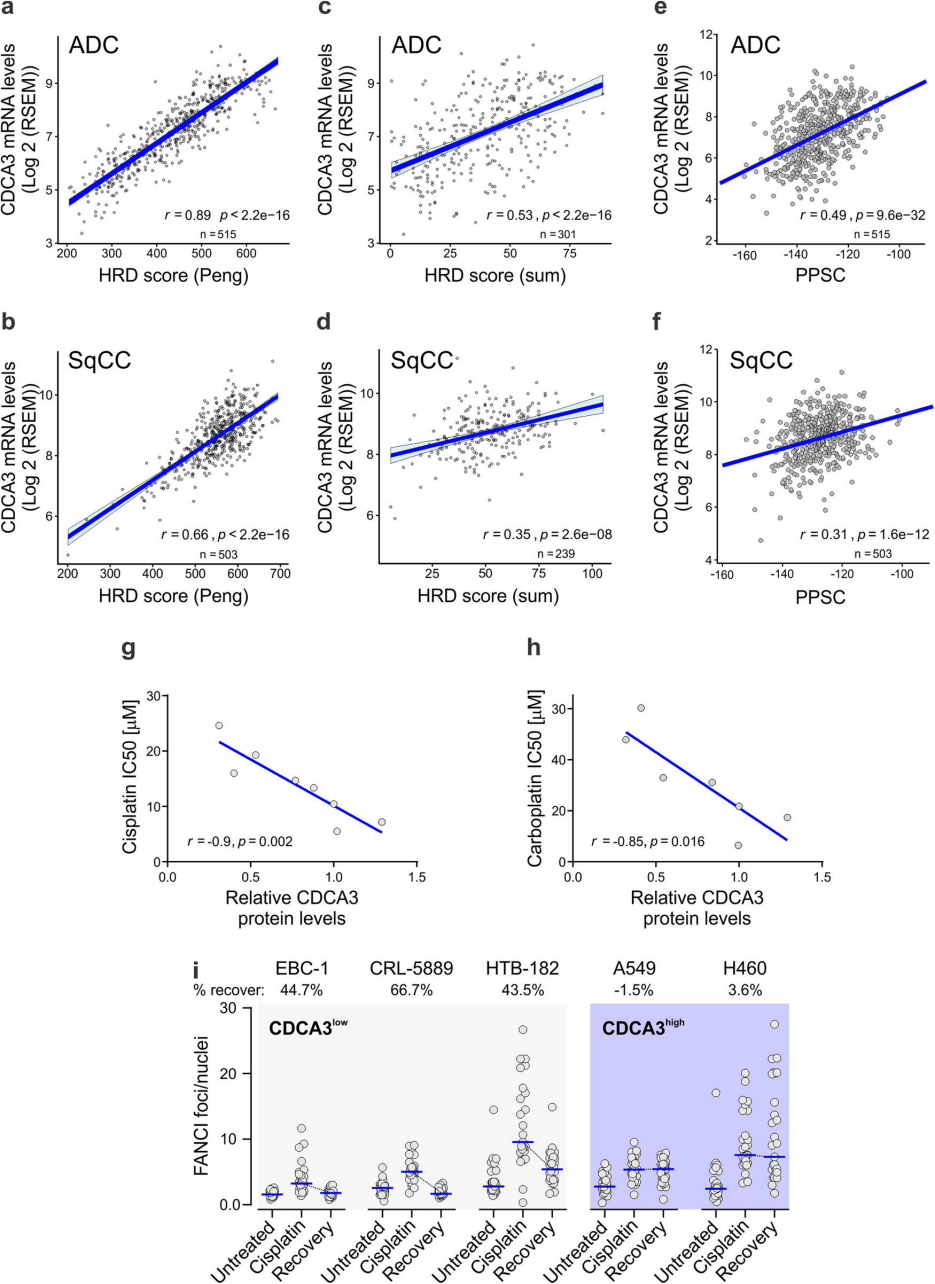
CDCA3 correlates with genome instability and cisplatin sensitivity. **a**–**f** Scatter plots showing linear regression analysis of The Cancer genome Atlas (TCGA) RNAseq datasets assessing the correlation between *CDCA3* levels and measures of genome instability (**a**–**d**) and predicted chemotherapy sensitivity (**e**, **f**) in NSCLC. *R* and *P* values determined according to Spearman’s rank correlation **a**, **b** Correlation with gene expression signature reflective of a homologous recombination deficiency (HRD) score in adenocarcinoma (ADC, **a**) and squamous cell carcinoma (SqCC, **b**). **c**, **d** Correlation in ADC (**c**) and SqCC (**d**) between *CDCA3* levels and HRD score calculated by the unweighted sum of three genomic scars, telomeric allelic imbalances, large scale genomic transitions and loss of heterozygosity. **e**, **f** Correlation with gene expression signature reflective of a pharmacogenomic predictor of pathologic complete response (pCR) to preoperative chemotherapy in ADC (**e**) and SqCC (**f**). **g** Scatter plot showing linear regression analysis assessing correlation between CDCA3 protein levels, determined by previous western blot analysis^18^ (see also Fig. 2a for endogenous CDCA3 protein levels), and in vitro cisplatin sensitivity which is represented by IC_50_ values. Cisplatin IC_50_ values calculated by plotting NSCLC cell viability for each of the escalating cisplatin doses (see Supplementary Fig. 1g). **h** Scatter plot showing linear regression analysis assessing correlation between CDCA3 protein levels and in vitro carboplatin sensitivity, represented by IC_50_ values. As per (**f**), IC_50_ values for all cell lines with the exception for H2228 were calculated from carboplatin dose response curves (see Supplementary Fig. 1g). *R* and *P* values determined according to Spearman’s rank correlation. **i** Beeswarm plots showing the foci count per nucleus of FANCI immunofluorescence microscopy for five NSCLC cell lines grouped by high or low CDCA3 protein levels (determined in **g**). Cells endogenously expressing high (A549 and H460 cells) versus low (EBC-1, CRL-5889 and HTB-182 cells) CDCA3 levels were untreated, cisplatin treated for 12 h (cisplatin) or cultured in fresh growth medium for 8 h following cisplatin treatment (recovery). Data points represent an average of FANCI foci/nuclei per field of view from a minimum of 800 nuclei (*n* = 13–16 fields; see Supplementary Fig. 1 for representative images). Blue lines indicate median values. Dotted black lines highlight change in foci count following recovery. Percent recovery calculated by difference between 100% and ratio of recovery/cisplatin expressed as a percentage.

To assess the utility of CDCA3 as a tool to identify platinum-based chemotherapy response in NSCLC, we evaluated available patient data for correlations with gene signatures or DNA-based measures of genome instability. Defective DNA repair is associated with improved response to DNA-damaging therapeutics such as platinum agents in lung cancer^19^ and other solid malignancies^20^. To this end, we undertook bioinformatics analyses of TCGA datasets to correlate relative *CDCA3* transcript levels with a homologous recombination deficiency (HRD) score in the ADC and SqCC NSCLC histologies. As shown in Fig. 1, *CDCA3* expression significantly correlated with a HRD score in ADC and SqCC disease determined firstly by a multigene signature representative of HR deficiencies^21^ (Fig. 1a, b) and the unweighted sum of three genomic scars, namely loss of heterozygosity, telomeric allelic imbalance and large scale state transitions^22^ (Fig. 1c, d). In each analysis, correlations between CDCA3 expression and HRD score were stronger in ADC disease. Further, *CDCA3* expression correlated with other features of genetic instability such as an increase in the number of gains or losses of chromosomal arms in both ADC and SqCC (see Supplementary Fig. 1a, b). We also identified that *CDCA3* levels were significantly higher in ADC and SqCC tumours with features of abnormal chromosome count, termed aneuploidy, versus diploid tumours (see Supplementary Fig. 1c, d). Consistently, *CDCA3* expression was significantly elevated in tumours exhibiting at least one whole-genome duplication in ADC and SqCC (see Supplementary Fig. 1e, f). Overall, *CDCA3* correlates with markers of genomic and chromosomal instability.

We next evaluated correlations between *CDCA3* and another multigene signature predictive of chemotherapeutic drug response, termed the pharmacogenomic predictor of sensitivity to chemotherapy (PPSC)^23^. While first applied in breast cancers to predict pathologic complete response (pCR) to chemotherapy^23^, this analysis has been used to assess NSCLC cases^24^ and shows utility for DNA-damaging therapeutics. In ADC (Fig. 1e) and SqCC (Fig. 1f) disease, CDCA3 expression positively correlated with the PPSC signature with respective Spearman correlation coefficients of *r* = 0.49 and *r* = 0.31.

**Fig. 2:**
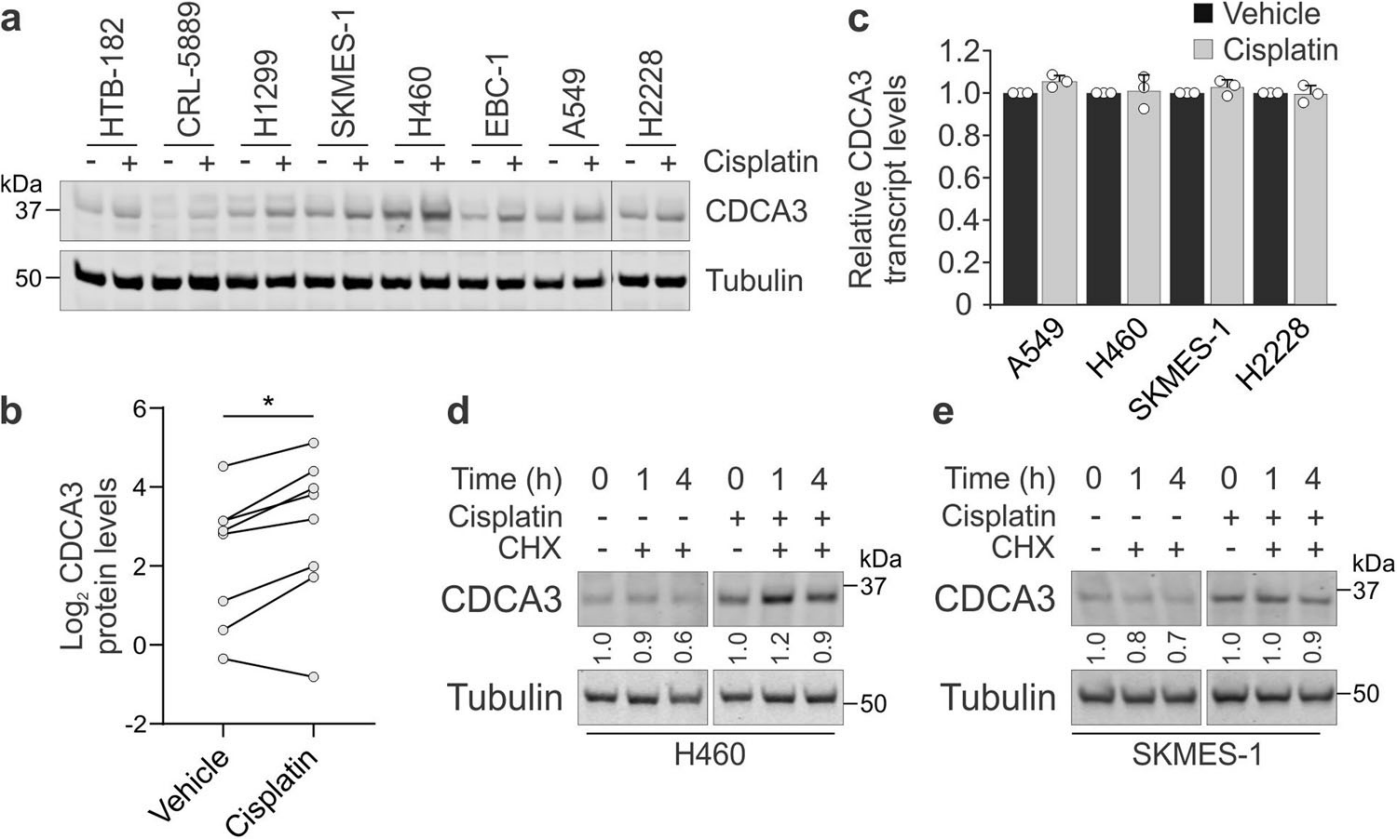
Platinum agents induce an upregulation of CDCA3 protein. **a** Representative endogenous CDCA3 western blot analysis from lysates of NSCLC cell lines treated in the absence or presence of cisplatin. Tubulin used as loading control. **b** Densitometry quantification of **a**, with dot points representing average log2 of relative CDCA3 levels from three independent experiments with lines connecting respective untreated and cisplatin treated cell lines (paired Student’s *t* test, *P = 0.0174). **c** Graphical representation of *CDCA3* transcripts from NSCLC cell lines treated with or without cisplatin determined using qRT-PCR. Comparative CT values normalised to housekeeping transcript 7SL and relative to untreated controls for each cell line. Mean ± SD from *n* = 3 in triplicate. **d**, **e** Endogenous CDCA3 western blot analysis of H460 (**d**) and SKMES-1 (**e**) lysates assessing protein turnover by treating with cycloheximide over 4 h in the presence or absence of cisplatin. Tubulin used as a loading control. Densitometry quantification of CDCA3 levels relative to 0 h indicated below CDCA3 blots.

Given the correlations in the clinical data, we evaluated the correlation between CDCA3 protein levels, determined by western blot analysis in our previous study^18^ (see also Fig. 2a for CDCA3 protein levels), with platinum agent potency (IC_50_ values) in a panel of NSCLC cell lines. Both cisplatin and carboplatin induced a dose-dependent reduction in cell viability across all cell lines tested (see Supplementary Fig. 1g). Consistent with our bioinformatics analyses, CDCA3 protein levels strongly correlated with the sensitivity of NSCLC cell lines to cisplatin (*r* = −0.9; Fig. 1g) and to carboplatin (*r* = −0.85; Fig. 1h), whereby CDCA3^high^ cell lines exhibited greatest cisplatin sensitivity. Of note, the CDCA3^high^ cell lines A549 and H460, versus three CDCA3^low^ cell lines, exhibited persistent nuclear foci of the DNA damage markers FANCI (Fig. 1i) and γH2AX (see Supplementary Fig. 1h) following an 8 h recovery from cisplatin exposure. Persistent DNA damage foci at the times tested, determined by immunofluorescence image analysis of high-throughput microscopy (see Supplementary Fig. 1i), point to a reduced DNA damage repair capacity. Collectively, these results suggest that CDCA3 expression correlates with DNA-based and functional measures of genome instability where CDCA3^high^ tumours are more sensitive to platinum agents.

### Platinum agents induce the upregulation of CDCA3 protein through evasion of APC/C^Cdh1^-mediated degradation

To explore mechanisms regulating CDCA3 levels, cisplatin treated NSCLC cell lines were examined for CDCA3 by western blot analysis. These data revealed that except for CRL-5889 cells, CDCA3 levels were upregulated ~1.5–2.5-fold in the majority of cell lines following 12 h of cisplatin treatment (Fig. 2a, b). *CDCA3* transcripts were also assessed by quantitative real-time PCR (qRT-PCR) in a smaller panel of NSCLC cell lines treated with cisplatin. As shown in Fig. 2c, cisplatin treatment did not significantly impact the levels of *CDCA3* transcripts in this small selection of cell lines.

We next asked whether platinum-induced stress could affect CDCA3 protein stability, thus yielding the increased levels of the protein. To do so, we chose the SKMES-1 and H460 cells, as model squamous and non-squamous NSCLC cell lines respectively, and also given that these cells have abundantly detectable CDCA3 protein levels suitable for protein half-life experiments. These cells were treated with cycloheximide to block protein synthesis in the presence or absence of cisplatin over the course of 4 h. Western blot analysis revealed that in the absence of cisplatin, CDCA3 levels were moderately reduced by ~30–50% in both H460 (Fig. 2d) and SKMES-1 cells (Fig. 2e), suggesting that the protein is being degraded in these cells. In contrast, CDCA3 protein turnover was markedly reduced in cisplatin treated NSCLC cells despite the presence of cycloheximide. Together, these data suggest that elevated CDCA3 levels induced by platinum result predominantly from increased protein stability and not enhanced transcription.

**Fig. 3:**
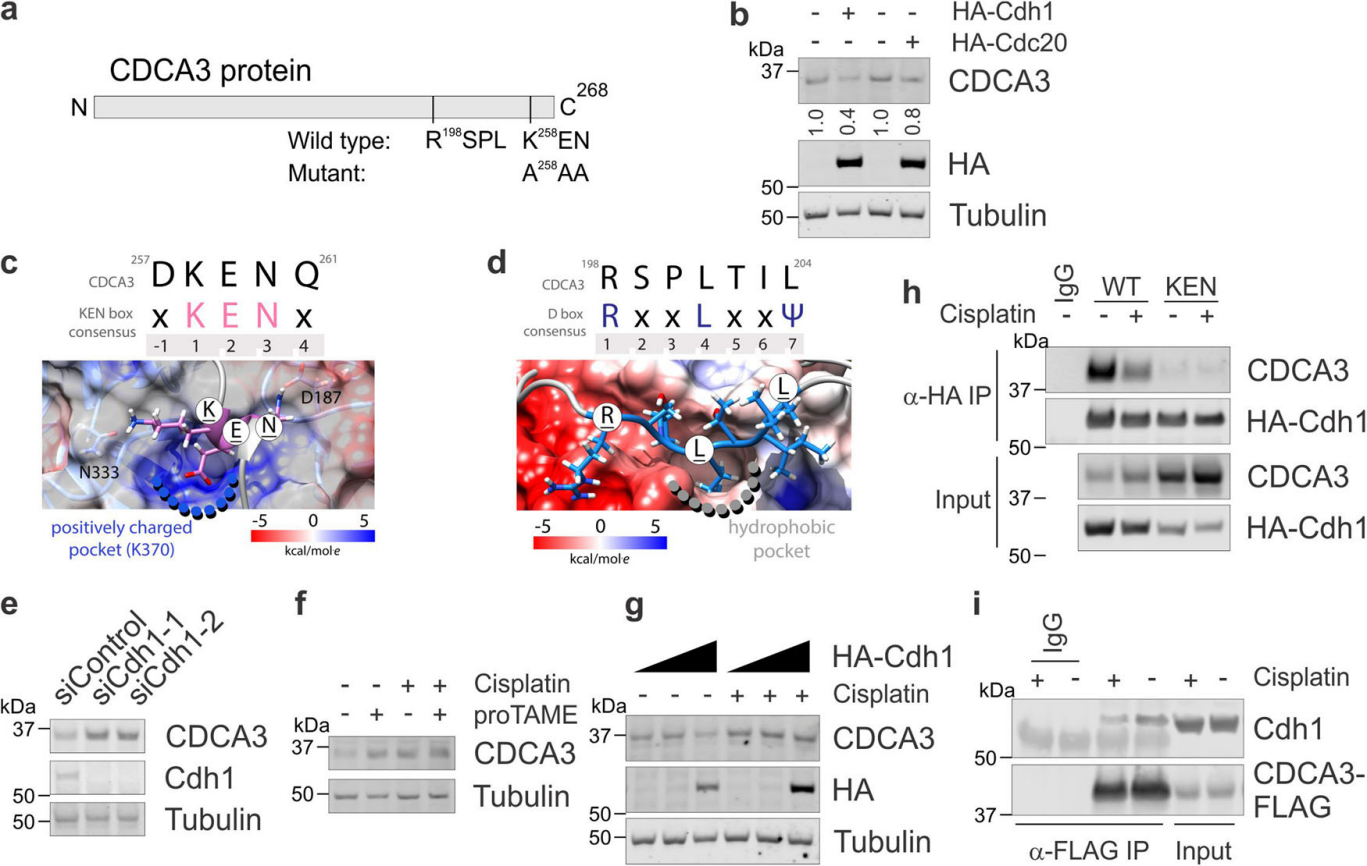
CDCA3 avoids APC/C^Cdh1^-mediated degradation following exposure to cisplatin. **a** Schematic of primary CDCA3 protein sequence listing C-terminal D-box (RxxL) and KEN box motifs. KEN box mutated to A^258^AA also indicated. **b** Endogenous CDCA3 western blot analysis of H460 cells ectopically expressing increasing levels of HA-Cdh1 or HA-CDC20. Tubulin used as loading control and HA probe to determine ectopic Cdh1 or CDC20 expression. **c**, **d** In silico models of the CDCA3 KEN box (**c**) and D-box (**d**) bound to Cdh1. Snapshots were taken after 300 ns of molecular dynamics simulation. Upper, CDCA3 protein sequence compared with consensus motifs. X = any amino acid, Ψ = aliphatic amino acid. Numbers indicate amino acid position within motif. *Lower*, colour scales highlighting the Columbic electrostatic potential at the surface of Cdh1 in these functional regions. **e** Western blot analysis of endogenous CDCA3 in control or Cdh1 depleted H460 cells. **f** Endogenous CDCA3 western blot analysis of H460 cells treated with cisplatin, the APC/C inhibitor proTAME or combination of both. **g** Western blot analysis of H460 cells expressing increasing levels of ectopic HA-Cdh1, as per Fig. 3b, treated with or without cisplatin. **h** Immunoprecipitation analysis of ectopic HA-Cdh1 with wild-type or KEN mutant CDCA3-FLAG from H460 cells treated with or without cisplatin. **i** Reciprocal immunoprecipitation analysis of ectopic CDCA3-FLAG with endogenous Cdh1 in H460 cells treated with or without cisplatin. All in vitro experiments are representative of three independent repeats.

To explore the possibility that CDCA3 levels are regulated by protein degradation, we searched the primary protein sequence of CDCA3 for known motifs recognised by ubiquitin ligases. As shown in Fig. 3a, CDCA3 contains a C-terminal KEN box motif starting at Lys258^8^ and a D-box R-X-X-L motif commencing at Arg198. Each of these motifs are necessary for substrate recognition by the co-activator proteins Cdh1 or Cdc20, which serve to recruit substrates for poly-ubiquitination by the large multi-subunit E3 ubiquitin ligase anaphase-promoting complex/cyclosome (APC/C) for degradation by the 26S proteasome. To confirm which of the APC/C co-activators is required to promote CDCA3 degradation in NSCLC, we ectopically expressed escalating levels of either HA-tagged Cdh1 or Cdc20 in H460 cells. For these experiments, H460 cells were used as a model NSCLC cell line suitable for transfections and as a cell line with readily detectable endogenous CDCA3 by western blot analysis. As shown in Fig. 3b, endogenous CDCA3 levels were markedly reduced upon increased ectopic Cdh1 expression and less so with ectopic Cdc20 expression, suggesting that Cdh1, consistent with earlier reports^8^, is the predominant co-activator required to regulate levels of CDCA3 in NSCLC. Noticeably, CDCA3 lacks a classical ABBA motif, which is reported to be required for Cdc20 binding^25^, perhaps defining the specificity for CDCA3 recognition by Cdh1 versus Cdc20.

We performed in silico analysis and molecular dynamics (MD) simulations to evaluate an interaction between CDCA3 and Cdh1. Firstly, PrDOS^26^ and PSIPRED^27^ protein sequence analyses suggested that CDCA3 is an intrinsically disordered protein which forms transient helical structures, particularly over sequences containing conserved motifs, including the KEN and D-box motifs (see Supplementary Fig. 2a, b). Our MD simulations confirmed the region containing the KEN motif (residues Gln254-Gln261) showed the presence of a transient helical structure (Fig. 3c) whereas the D-box motif did not converge to any prominent secondary structure within the timescale of the simulation (Fig. 3d). The KEN box of CDCA3 formed strong hydrogen bonds with Asn333, Asn335, Gln401, Thr377, Arg445, Phe188, Asp187, Tyr189 and Tyr420 of Cdh1. The D-box of CDCA3 engaged in hydrophobic interactions and formed hydrogen bonds with Asp180, Glu465 and Val219 of Cdh1. Indeed, our molecular model of CDCA3 and Cdh1 highlights that residues of CDCA3 interact with regions of Cdh1 that are consistent with other known Cdh1 interactants^28^.

To experimentally confirm that Cdh1 modulates CDCA3 levels, H460 cells were depleted of Cdh1 using two independent siRNAs. Western blot analysis revealed that knockdown of Cdh1 resulted in elevated CDCA3 protein levels, further suggesting Cdh1 is the co-activator that regulates cellular CDCA3 levels (Fig. 3e). Consistently, treating H460 cells with proTAME, a cell permeable APC/C inhibitor^29^, in the presence and absence of cisplatin induced the upregulation of CDCA3 protein (Fig. 3f). In addition, unlike in untreated cells, increased ectopic Cdh1 expression did not reduce endogenous CDCA3 protein levels in cisplatin treated H460 cells (Fig. 3g). These data further indicate that CDCA3 protein levels are regulated in an APC/C^Cdh1^-dependent manner, particularly following platinum-induced stress.

To investigate the impact on binding to Cdh1, we performed immunoprecipitations between ectopic HA-Cdh1 and either CDCA3-WT-FLAG or the KEN box mutant (listed in Fig. 3a) that were expressed in H460 cells either treated with or without cisplatin. As shown in Fig. 3h, while loss of the KEN box completely abrogated binding to Cdh1, cisplatin treatment markedly reduced the association between CDCA3 and Cdh1. Reciprocal immunoprecipitation analysis indicated that platinum-induced stress reduced the association between ectopic CDCA3-FLAG and endogenous Cdh1 in H460 cells (Fig. 3i). These data highlight that levels of CDCA3 are regulated by the APC/C^Cdh1^, particularly following platinum-induced stress.

### Phosphorylation of CDCA3 by CK2 promotes an interaction with Cdh1 and degradation by the APC/C^Cdh1^

**Fig. 4:**
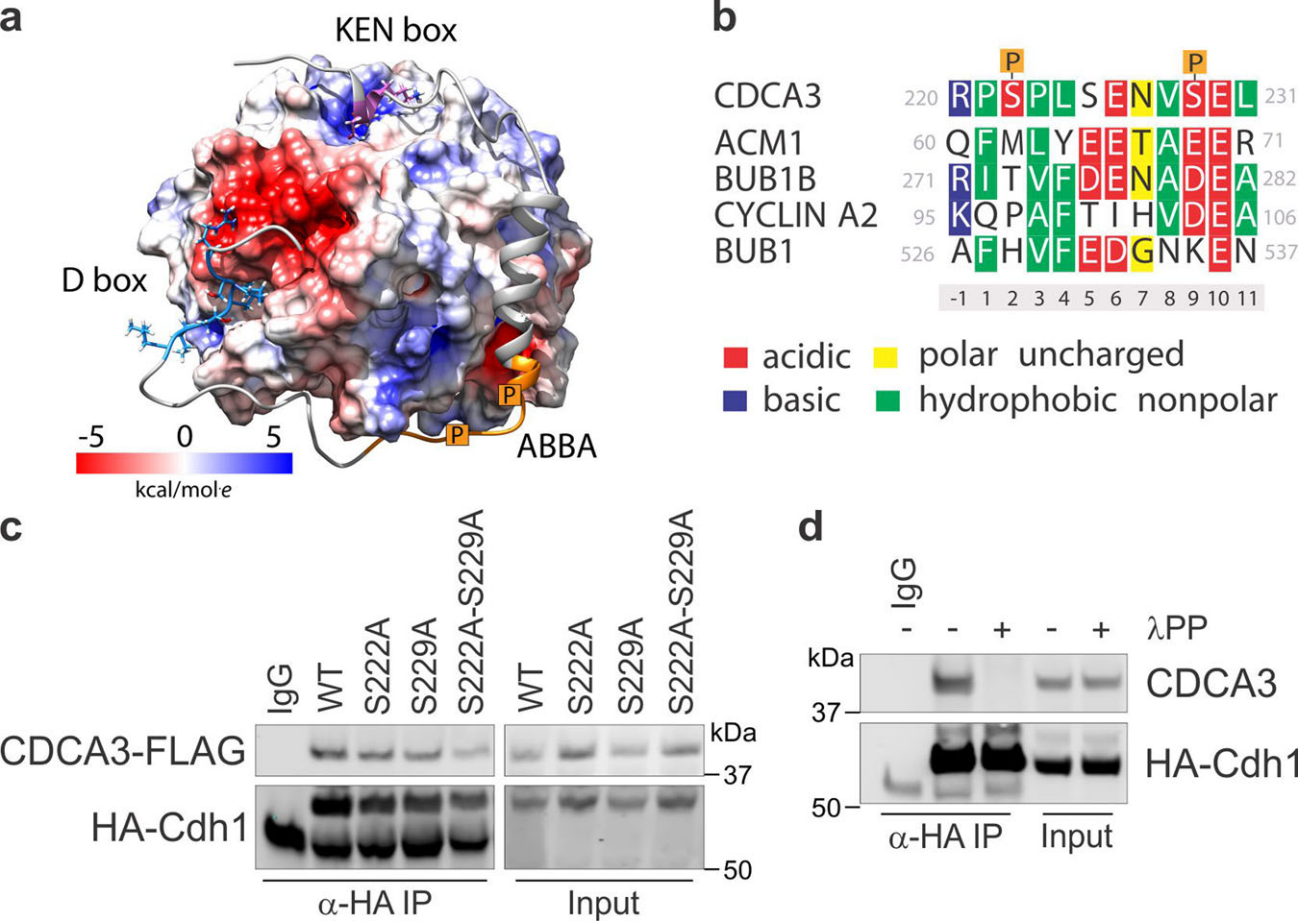
Phosphorylation of CDCA3 promotes an interaction with Cdh1. **a** Molecular simulations of CDCA3 C-terminal region (Ser193-Glu267) with Cdh1 highlighting contribution of CDCA3 phosphorylation to an interaction with Cdh1. CDCA3 D-box, KEN box and phosphorylation sites (in orange) indicated. Lower, colour scale indicating the Columbic electrostatic potential for the entire surface of Cdh1. **b** Sequence alignment (upper) of CDCA3 region containing phosphorylated Ser222 and Ser229 with characterised ABBA motif containing proteins. Numbers below the sequences indicate amino acid position with respect to the identified motif. Lower, a colour key describing the charge and hydrophobicity used to categorise residues in sequence alignment. **c** Immunoprecipitation analysis of ectopic HA-Cdh1 with wild-type or phosphorylation site mutants of CDCA3-FLAG expressed in H460 cells. **d** Immunoprecipitation analysis of ectopic HA-Cdh1 with endogenous CDCA3 from H460 cell lysates either untreated or treated with lambda phosphatase (λPP) to remove phosphate moieties from proteins. All in vitro experiments are representative of three independent repeats.

To define molecular mechanisms regulating the levels of CDCA3, we further investigated the MD simulations of CDCA3 bound with Cdh1. For our modelling, we focused on the C-terminal region of CDCA3 containing the KEN and D-boxes which were bound to their respective regions within Cdh1. As shown in a representative snapshot from the simulation, CDCA3 formed a transient helical region from residues Glu230 to Lys244 and associated with a corridor of positive charges within Cdh1 (Fig. 4a). In particular, Ser222 and Ser229 when phosphorylated and with a net negative charge, interact within a positively charged cleft on the surface of Cdh1. Indeed, phosphorylation of these residues have been detected in phosphoproteome-wide screens^30–32^. Sequence alignment of this region of CDCA3 highlights a similarity with known APC/C substrates, including ACM1, BUB1B, Cyclin A2 and BUB1^25^, each containing ABBA motifs (Fig. 4b). Despite low sequence homology, charged or hydrophobic residues exist in similar positions between each of these APC/C substrates, raising the possibility that this non-canonical ABBA-like motif within CDCA3 aides an association with Cdh1.

We verified our simulations by immunoprecipitating HA-Cdh1 with either CDCA3-WT-FLAG or mutant forms of CDCA3-FLAG where the serine residues were individually, or both substituted with alanine residues. While individual loss of either Ser222 or Ser229 alone did not reduce an association with Cdh1, dual loss of both residues markedly reduced an association between CDCA3 and Cdh1 in H460 cells (Fig. 4c). Consistently, lambda phosphatase treatment of H460 cell lysates to remove phosphate moieties, abrogated the association of ectopic HA-Cdh1 and CDCA3-WT-FLAG by immunoprecipitation (Fig. 4d). These data suggest that post-translational modification of CDCA3 at Ser222 or Ser229 generates a non-canonical ABBA-like motif that, alongside the classical KEN and D-box motifs, is required for an efficient association between CDCA3 and APC/C^Cdh1^.

**Fig. 5:**
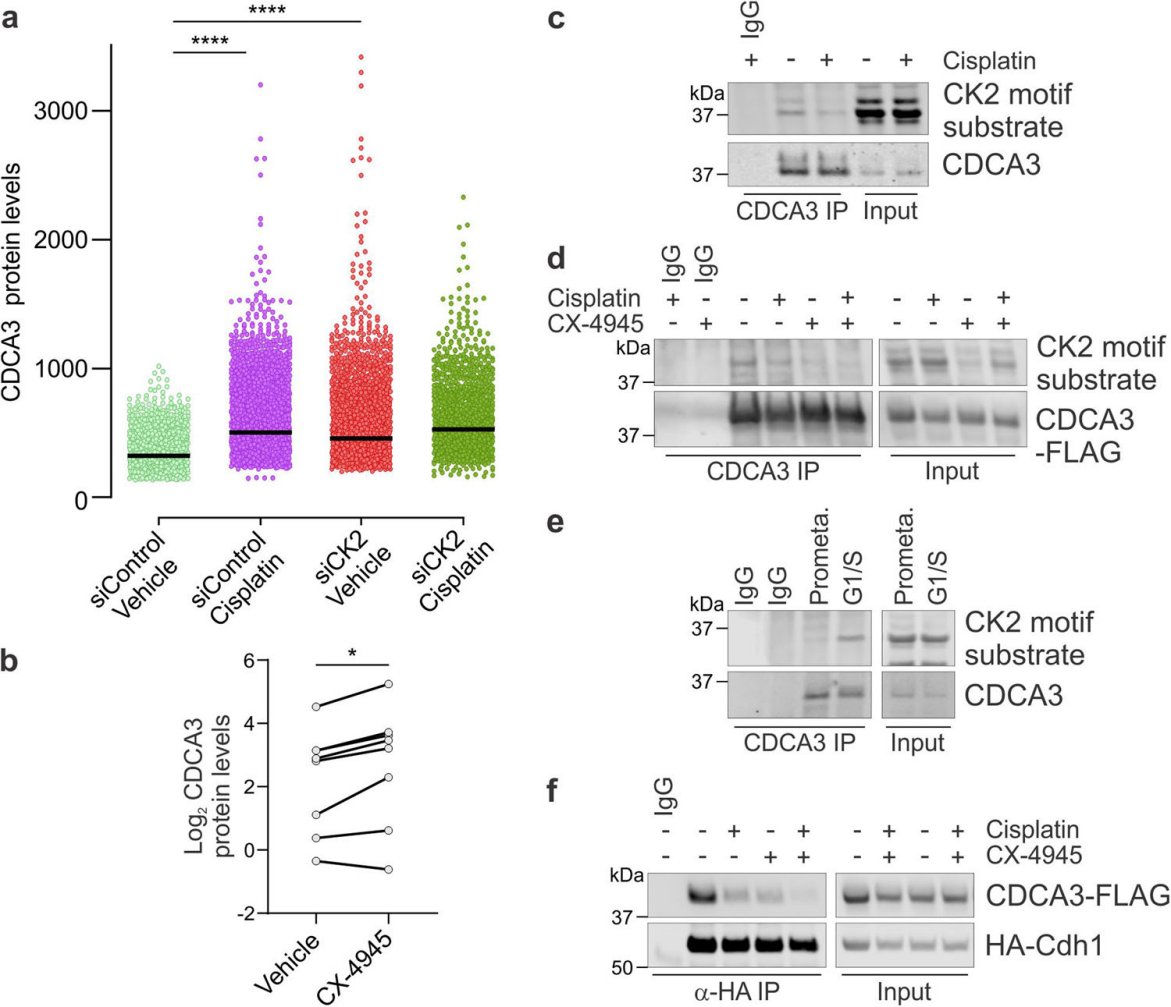
Phosphorylation of CDCA3 by CK2 promotes its degradation by the APC/C^Cdh1^. **a** Beeswarm plot showing CDCA3 levels assessed in control or CK2 depleted H460 cells that were treated with or without cisplatin. CDCA3 levels determined by analysis of high throughput immunofluorescence microscopy images. Data points represent CDCA3 levels within individual cells from a minimum of 1200 cells (see Supplementary Fig. 3a). Horizontal lines indicate median values (unpaired Student’s *t* test, ****P = < 0.0001). **b** Densitometry quantification of western blot analysis in Supplementary Fig. 3b, with dot points representing average log2 of relative endogenous CDCA3 levels from NSCLC cell lines treated with or without CX-4945. Representative of three independent experiments with lines connecting respective untreated and cisplatin treated cell lines (paired Student’s *t* test, *P = 0.0126). **c** Immunoprecipitation analysis of endogenous CDCA3 to assess the CK2-mediated phosphorylation of CDCA3 in untreated or cisplatin treated H460 cells. **d** Immunoprecipitation analysis of ectopic CDCA3 from H460 cell lysates to assess the impact of cisplatin, CX-4945 or combination of both upon CK2-mediated phosphorylation of CDCA3. **e** Immunoprecipitation analysis to assess CK2-mediated phosphorylation of enriched endogenous CDCA3 from nocodazole arrested H460 cells in prometaphase (prometa.) or H460 cells arrested at the G1/S cell cycle boundary by double thymidine block. **c**–**e** CK2 phosphorylation assessed using antibody specific for CK2 phosphorylation motifs within substrates. **f** Immunoprecipitation analysis of ectopic HA-Cdh1 with ectopic wild-type CDCA3-FLAG from H460 cells treated with cisplatin, CX-4945 or a combination of both. All in vitro experiments are representative of three independent repeats.

To identify kinases responsible for the phosphorylation of, and hence regulation of CDCA3 levels, we performed an siRNA-based screen for 38 different kinases and assessed the levels of CDCA3 using immunofluorescence and high throughput microscopy. Of the 38 different kinases (see Supplementary Fig. 3a), only depletion of the casein kinase 2 (CK2) catalytic subunits, α (CSNK2A1) and ά (CSNK2A2), yielded a ~1.5-fold upregulation of CDCA3 protein levels compared with siRNA control transfected cells. Consistently, immunofluorescence analysis indicated that CDCA3 levels were elevated in CSNK2A1-depleted H460 cells treatment in the absence or presence of cisplatin (Fig. 5a). CK2 depletion resulted in CDCA3 upregulation to a degree comparable with cisplatin treatment alone (Fig. 5a). We sought to verify the siRNA experiments by using a pharmacological approach. CX-4945 is a potent small molecule inhibitor of CK2 that has undergone clinical testing^33–36^. As shown in Fig. 5b and see Supplementary Fig. 3b, 24 h treatment of CX-4945 induced a ~1.3–2.3-fold upregulation of CDCA3 protein in all NSCLC cell lines, with the exception of CRL-5889 cells. To confirm the possibility that CDCA3 is a CK2 substrate, we probed immunoprecipitated endogenous CDCA3 from control or cisplatin treated H460 cells with an anti-phospho-CK2 substrate motif specific antibody. As shown in Fig. 5c, we detected immunoreactivity against CDCA3 with the CK2 substrate antibody which was reduced by ~60% following cisplatin treatment. Confirming the specificity of the CK2 substrate antibody, immunoreactivity against immunoprecipitated CDCA3 was prevented by CX-4945 treatment, both alone and when combined with cisplatin (Fig. 5d).

Given CDCA3 levels are lowest in the G1 cell cycle phase, reportedly due to degradation by the APC/C^Cdh1^ (ref. ^8^), we next assessed whether the CK2 phosphorylation status of immunoprecipitated CDCA3 varied across cell cycle phases. Phospho-CK2 immunoreactivity was detected in H460 cells arrested at the G1/S boundary by double thymidine treatment but not in mitotic arrested nocodazole treated cells (Fig. 5e). These data point to the possibility that CK2 phosphorylation might contribute toward regulating CDCA3 protein levels across the unperturbed cell cycle.

To examine the impact of CK2 phosphorylation upon CDCA3 association with Cdh1, immunoprecipitations between ectopic HA-Cdh1 and CDCA3-WT-FLAG were performed in the absence or presence of CX-4945, cisplatin or combination of both. As shown in Fig. 5f, like cisplatin, CX-4945 reduced the association between CDCA3 and Cdh1. Together, these data suggest CK2 plays a key role in regulating the levels of CDCA3, particularly following platinum-induced stress, whereby phosphorylation promotes the interaction between CDCA3 and Cdh1.

### Combination of CK2 inhibitors with cisplatin show greater effectiveness in CDCA3^low^ NSCLC cells

**Fig. 6:**
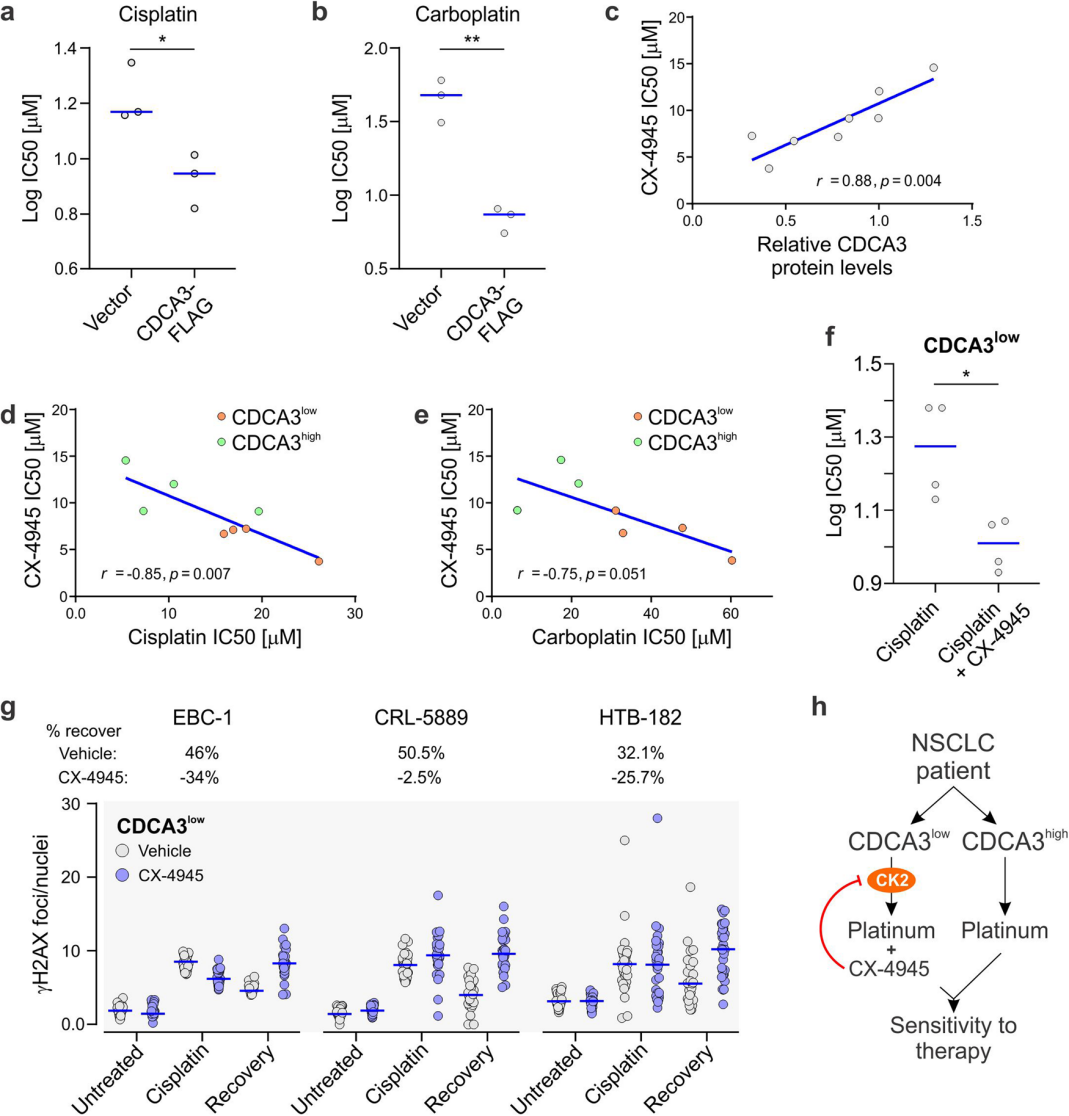
Combining CK2 inhibitors with cisplatin in CDCA3^low^ NSCLC. **a** Beeswarm plots showing the impact of ectopic CDCA3 overexpression in CDCA3^low^ NSCLC cell lines upon cisplatin dose responses. Blue line indicates median cisplatin IC_50_ value. (unpaired Student’s *t* test, *P = 0.0234). **b** Beeswarm plots showing the impact of ectopic CDCA3 overexpression in CDCA3^low^ NSCLC cell lines upon carboplatin dose responses. Blue line indicates median cisplatin IC_50_ value. (unpaired Student’s *t* test, **P = 0.0012). **a**, **b** The CDCA3^low^ cell lines used include EBC-1, CRL-5889 and HTB-182 cells. **c** Scatter plot showing linear regression analysis assessing correlation between CDCA3 protein levels, and in vitro CX-4945 sensitivity which is represented by IC_50_ values, as per Fig. 1g. CX-4945 IC_50_ values calculated dose responses indicated in Supplementary Fig. 4. Data points represent NSCLC cell lines. *R* and *P* values determined according to Pearson correlation. **d** Scatter plot assessing correlation between cisplatin and CX-4945 IC_50_ sensitivities with NSCLC cell lines with high (green) and low (orange) CDCA3 indicated. **e** Scatter plot assessing correlation between carboplatin and CX-4945 IC_50_ sensitivities with NSCLC cell lines with high (green) and low (orange) CDCA3 indicated. **d**, **e** Linear regression determined according to Pearson correlation with *R* and *P* values displayed. **f** Beeswarm plots showing the impact of CX-4945 IC_25_ concentrations upon cisplatin dose responses in NSCLC cell lines with low CDCA3 levels. Blue line indicates median cisplatin IC_50_ value. (unpaired Student’s *t* test, *P = 0.0138). **g** Beeswarm plots showing γH2AX foci count per nucleus determined from high throughput immunofluorescence microscopy of EBC-1, CRL-5889 and HTB-182 NSCLC cell lines with low CDCA3 protein levels (determined in Fig. 1g). γH2AX foci counts were determined in cell lines exposed to the presence (blue data points) or absence (grey data points) of CX-4945 and either cisplatin treated for 12 h (cisplatin) or untreated. Foci counts were also determined in recovering cells following treatment with cisplatin alone or in combination with CX-4945 following cisplatin treatment (recovery). Data points calculated as per Fig. 1h from *n* = 13–16 fields of view. Blue lines indicate median values. Percent recovery calculated by difference between 100% and ratio of recovery/cisplatin expressed as a percentage. **h** Schematic representing proposed strategy to use CDCA3 as a potential companion diagnostic for CX-4945 to improve the sensitivity of NSCLC tumours to platinum-based chemotherapy.

Our bioinformatics and in vitro analyses uncovered that CDCA3^high^ NSCLC is more responsive to chemotherapy and platinum agents than CDCA3^low^ tumours. To confirm this, we ectopically overexpressed CDCA3 in EBC-1, CRL-5889 and HTB-182 cells which are endogenously CDCA3^low^. By increasing CDCA3 protein levels in each of these cell lines (Supplementary Fig. 4a), the median cisplatin (Fig. 6a) and carboplatin (Fig. 6b) IC_50_ values were reduced ~2- and ~6-fold respectively. These data suggest that increasing CDCA3 levels might improve platinum agent potency.

Given levels of CDCA3 protein are regulated by platinum in a CK2-dependent manner, we sought to determine whether CDCA3 had companion diagnostic potential in in vitro NSCLC models. To explore this, we evaluated the correlation of CDCA3 protein levels with CX-4945 potency (IC_50_ values) in a panel of eight cell lines. CX-4945 reduced cell viability in a dose-dependent manner across all cell lines tested (see Supplementary Fig. 4b). CDCA3 levels strongly correlated with sensitivity to CX-4945 (*r* = 0.88), whereby CDCA3^low^ cell lines showed greater sensitivity (Fig. 6c). We next assessed the correlation between cisplatin and carboplatin potencies with CX-4945 potencies across all cell lines tested. As shown in Fig. 6b, sensitivity to cisplatin was inversely proportional to CX-4945 sensitivity with a strong negative correlation (*r* = −0.85). Although not statistically significant, carboplatin sensitivity was also inversely proportional to CX-4945 sensitivity (*r* = −0.75, Fig. 6e). By stratifying each cell line based on median CDCA3 expression, we identified that for both cisplatin and carboplatin potency correlations, CDCA3^low^ cells and CDCA3^high^ cells grouped separately, with the sole exception of H1299 cells in Fig. 6d. Based on these analyses, we evaluated the combination of cisplatin with respective IC_25_ CX-4945 concentrations in the CDCA3^low^ cell lines, which are less sensitive to cisplatin than CDCA3^high^ cells (Figs. 1g and 6d). As shown in Fig. 6f, CX-4945 significantly enhanced median cisplatin potency from ~19 µM to ~10 µM. We next assessed whether CK2 inhibition might impact DNA repair capacity in the CDCA3^low^ EBC-1, CRL-5889 and HTB-182 cell lines by examining γH2AX foci as per Fig. 1i. As shown in Fig. 6g, combining CX-4945 with cisplatin increased the level of persistent γH2AX foci following drug recovery versus cisplatin treatment and recovery alone in all three cell lines. CX-4945 treatment alone did not induce γH2AX foci in these cell lines (Fig. 6g). These findings suggest CX-4945 sensitises NSCLC cells to cisplatin by reducing DNA repair capacity, particularly in those tumours selected based on CDCA3 expression.

## Discussion

Predictive biomarkers of platinum response are necessary to improve the clinical management of lung cancer patients undergoing therapy. Our findings point to the possibility of exploiting CDCA3 expression to select NSCLC patients for platinum-based therapy (Fig. 6h). We document that in in vitro analyses and available patient data, CDCA3 expression correlates with genomic and functional measures of genome instability and platinum sensitivity in vitro. Functionally, CDCA3 has not been directly linked with maintenance of genome stability. However, CDCA3 has been identified in network analysis to contribute toward enabling tumours to cope with high levels of genome stability^37^. This is in line with our observation that cisplatin-induced DNA damage causes an upregulation of CDCA3 protein via evasion of APC/C^Cdh1^-mediated degradation (Figs. 2 and 3), suggesting that upregulation of CDCA3 expression is symptomatic of, and not causal of genome instability. However, upregulation of CDCA3 levels by ectopic expression resulted in enhanced sensitivity to cisplatin and carboplatin (Fig. 6a, b). Further work is required to determine whether modulating CDCA3 protein levels directly impacts genome stability. Despite responding to ICL DNA damage, we demonstrate that basal CDCA3 expression correlates with genome stability and platinum sensitivity (Fig. 1). It is worth noting that while our study focussed on sensitivity or an initial response to cisplatin, basal CDCA3 expression does not make any conclusions about likelihood of acquired platinum resistance. It is possible that some of the cell lines, perhaps those CDCA3^low^ cell lines, are representative of innate resistance but further studies are required. Nonetheless, our findings highlight the potential for basal CDCA3 levels as a screening tool at initial biopsy for treatment naïve NSCLC patients, in a process similar to the current immunotherapy biomarker PD-L1^38^. Although requiring tissue biopsy, there are advantages to this approach versus other multimodal genomic or gene signature biomarkers^39^. These include not being reliant upon specialist equipment, cost and ease of implementation^40^. Moreover, given our prior validation of immunohistochemistry staining for CDCA3^18^, this method would be ideal to detect protein levels in tissue biopsies. However, further studies are necessary to define the low versus high expression staining parameters and to assess the predictive power for these CDCA3 expression parameters for determining therapy responsiveness in the clinical setting.

Our findings also point to the possibility that CDCA3 expression has companion diagnostic potential for the drug CX-4945, to improve cisplatin sensitivity in NSCLC (Fig. 6e). As a potent CK2 inhibitor, CX-4945 is currently under phase I/II clinical trial for cholangiocarcinoma patients in combination with a chemotherapy regimen containing cisplatin (clinicaltrials.gov identifier: NCT02128282). CK2 is a constitutively active tetrameric serine/threonine kinase, consisting of two catalytic (α and/or ά) and two regulatory (β) subunits, with a diversity of roles in cell cycle control, regulation of transcription, DNA repair and survival^41–43^. We document that CK2 activity plays a key role in regulating CDCA3 levels by modulating its degradation by APC/C^Cdh1^. Our findings highlight that, like other CK2 substrates, including those involved in maintaining genome stability^44–47^, phosphorylation promotes protein-protein interaction, in this case between CDCA3 and Cdh1. It is worth noting that the CDCA3 phosphorylation sites Ser222 and Ser229 deviate from the S/T-X-X-D/E CK2 consensus motif. However, CDCA3 does contain negatively charged residues at positions *n* + 1 for Ser229 (Glu230) and *n* + 4 for both serines (Glu256 and Glu231) which are also observed in other substrates^48^. Moreover, immunoprecipitated CDCA3 is immunoreactive with a CK2 phosphorylation specific antibody that is sensitive to CX-4945 (Fig. 5). Indeed, with these phosphorylation sites, we propose to have identified a novel non-canonical ABBA-like motif, which aligns electrostatically with other characterised ABBA motifs, promoting an interaction with Cdh1 (Fig. 4). Our findings suggest that this ABBA-like motif is dynamically regulated, given cisplatin-induced reduced phosphorylation of CDCA3 by CK2 causing a dissociation between CDCA3 and Cdh1. Although CK2 is active in DNA repair and in each cell cycle stage, it is likely that Ser222 and Ser229 are subject to regulation by unidentified phosphatases, particularly during DNA damage and mitosis (Fig. 5). Nonetheless, in those cells with low CDCA3 levels, possibly due to CK2 promoted degradation, CK2 inhibition sensitises NSCLC to platinum. While CX-4945 enhances chemotherapy in other solid malignancies^49,50^, our findings indicate that in NSCLC, a strategy to first select tumours based on CDCA3 expression would enhance CX-4945 potency and sensitivity to platinum agents. Importantly, our findings lay the foundation for further testing in patient-derived disease models and future retrospective and prospective patient cohorts prior to clinical trial.

## Methods

### Ethical compliance

All human data was obtained from public resources with non-identifiable patients providing informed consent according to TCGA Ethical Board regulations (https://www.cancer.gov/about-nci/organization/ccg/research/structural-genomics/tcga/history/policies). All methods were also performed in accordance with relevant guidelines and regulations and approved by Queensland University of Technology (approval number 1900000269).

### Antibodies, reagents and mammalian expression constructs

The CDCA3 antibody (HPA026587), monoclonal FLAG M2 antibody (F1804) and α-Tubulin antibody (T9026) were purchased from Sigma Aldrich. Antibodies against the HA tag (#3724) and phospho-CK2 substrate motif (#8738) were purchased from Cell Signaling Technology (Genesearch, Australia). The Cdh1 (ab77885) and γH2AX (ab26350) antibodies were from Abcam. The FANCI antibody (A301-254A) was from Bethyl Laboratories, Inc. 4′-6-diamidino-2-phenylindole (DAPI) was from Life Technologies and Complete EDTA-free protease inhibitor mixture was from Roche Applied Sciences. Donkey anti-rabbit and anti-mouse Alexa Fluor 488 antibodies were purchased from Life Technologies. CX-4945 was purchased from Selleck Chemicals Llc and proTAME was purchased from BostonBiochem. All other reagents were from Sigma-Aldrich except where noted. Mammalian expression construct for HA-Cdh1 was a gift from Marc Kirschner (Addgene plasmid #11596^51^) and the CDCA3-FLAG expression construct was generated by subcloning the full-length CDCA3 sequence in pcDNA3.1+ using the BamHI and EcoRI restriction enzyme sites. N-terminal consensus kozak sequence and C-terminal triple FLAG sequence were introduced using PCR.

### Cell culture, transfections and cell treatments

All NSCLC cell lines were obtained from the American Type Culture Collection (ATCC) and maintained in RPMI-1640-medium + l-glutamine (Life Technologies), supplemented with 10% foetal bovine serum (FBS, Sigma Aldrich). All cell lines were cultured at 37 °C in a humidified 5% CO_2_ atmosphere.

For transfections of the customised siRNA library, Lipofectamine RNAimax was used (Life technologies). The customised Silencer Select human kinase siRNA library (Cat. No. 4392425, Life Technologies) contained 62 lyophilised siRNAs targeting 38 different human kinases (see Supplementary Fig. 3a for listing of kinases). siRNAs in a 96 well were rehydrated according to manufacturer’s instructions and transfected into adherent H460 cells in glass bottom, black walled immunofluorescence 96-well plates. Scrambled Silence Select siRNAs were used as transfection controls (Life Technologies). Lipofectamine RNAimax was also used for transfection of individual Silencer Select pre-designed siRNAs targeting *FZR1* (Cdh1 protein) and *CSNK2A1* (Life Technologies). siCdh1-1 (ID: s27991); siCdh1-2 (ID: s27993); and siCK2 (DI: s3637). For expression construct transfection, FuGene HD transfection reagent was used (Promega Corporation).

For in vitro drug treatments prior to western blot or immunoprecipitation analysis, cells were treated with cisplatin (5 µM) or CX-4945 (5 µM) for 12 h or proTAME (10 µM) for 6 h. For protein half-life experiments, cells were treated with cycloheximide (70 µM) for 4 h in the presence or absence of cisplatin (5 µM). To block cells in prometaphase, cells were treated with either nocodazole (0.1 µg/mL) for 16 h. To block cells at the G1/S cell cycle boundary, cells were treated with thymidine (2 mM) for 16 h, followed by recovery in fresh culture media for 8 h before a second 16-h thymidine treatment.

### Collection of lysates, immunoprecipitation and western blot analyses

For whole cell lysate collection, cells were washed with phosphate-buffered saline and lysed in lysis buffer (50 mM HEPES (pH 7.5), 150 mM KCl, 5 mM EDTA, 0.05% IGEPAL CA-630 (v/v), 1x protease inhibitor cocktail (Roche) and 1x phosphatase inhibitor cocktail (Cell Signaling Technology)). Following sonicated and centrifugation, total protein yield was determined by Bicinchoninic Acid (BCA) Protein assay (Sigma Aldrich). Total protein (20 µg) samples were denatured in 1x Laemmli Buffer supplemented with 8% β-mercaptoethanol for 5 min at 80 °C.

For immunoprecipitation, protein samples were prepared with 400 µg protein in 400 µl of lysis buffer. Lysates were incubated overnight with 3 µg of FLAG antibody, HA antibody or CDCA3 antibody overnight at 4 °C. Following incubation, lysates were incubated with protein A or G Dynabeads pre-equilibrated with lysis buffer (Invitrogen). The Dynabeads were denatured using 2x Laemmli sample buffer supplemented with 8% β-mercaptoethanol for 5 min at 80 °C.

Samples were separated on Bolt 4–12% Bis-Tris Plus pre-cast gels (Life Technologies) and transferred onto nitrocellulose membrane (GE Healthcare Life Sciences) using the semi-dry transfer Novex system (Life Technologies). Membranes first blocked using Odyssey blocking buffer (Li-Cor) were incubated with primary antibody overnight at 4 °C in a 1:1 solution of Odyssey blocking buffer and PBS-T. All primary antibodies were used at a dilution of 1:1000 except for antibodies targeting CDCA3 (1:800) and tubulin (1:5000). Following incubation, membranes were washed with PBS-T and incubated with appropriate secondary antibodies and imaged using the Li-Cor Odyssey system (Li-Cor). Images were acquired and subject to densitometry analysis.

### Quantitative real time PCR

Total RNA was isolated using an Isolate II RNA mini kit (Bioline). Equal amounts of RNA (1 µg), quantified using a Nanodrop Lite spectrophotometer (Thermo Fisher Scientific), were reverse transcribed to generate cDNA using the Tetro cDNA Synthesis kit (Bioline). *CDCA3* (forward primer 5′-GGACCCTGAGACTCCCAGAT-3′ and reverse primer 5′-GCCGCTTACCCTGTCGTAG-3′) and *7SL* transcript levels (forward primer 5′-ATCGGGTGTCCGCACTAAGTT-3′ and reverse primer 5′-CAGCACGGGAGTTTTGACCT-3′) were determined using SYBR Green (Thermo Fisher Scientific) on a ViiA7 RT-PCR system (Thermo Fisher Scientific). *CDCA3* levels were normalised to *7SL* levels and analysed using the comparative CT method.

### Immunofluorescence and high content microscopy

Cells were seeded at 1.0 × 10^4^ to 1.2 × 10^4^ cells per well on a glass-base 96-well plate (Corning) at and left to adhere for 12 h overnight before drug treatment. For treatments, cells were exposed to IC_50_ concentrations of cisplatin for 12 h either alone or in the presence of IC_50_ concentrations of CX-4945. For recovery experiments, drug treated cells were washed with PBS and cultured in fresh media for 8 h. Cells were fixed with 4% paraformaldehyde (PFA) in PBS for 20 min at room temperature (RT) and permeabilised with 0.1% Triton X-100 in PBS for 5 min at ambient temperature. The cells were subsequently blocked with 2% Donkey serum in PBS for 30 min at ambient temperature. Primary antibodies were diluted in 0.5% Donkey serum in PBS and incubated overnight at 4 °C. Antibodies targeting FANCI, γH2AX and CDCA3 were used at dilutions of 1:100, 1:1000 and 1:300 respectively. Fluorescent secondary antibodies conjugated with Alexa Fluor® dye were diluted in 0.5% Donkey serum in PBS and incubated for 1 h at RT (1:600 dilution). Following this, the cells were stained with Hoechst 33342 stain diluted in PBS (final concentration of 1 µg/mL) and incubated at RT for 5 min before imaging. Images were collected on an InCell Analyzer 6500 high content microscopy imaging system (GE Healthcare Life Sciences). Images were analysed using the CellProfiler software v3.1.9. FANCI or γH2AX foci were reported as foci per nuclei per field of view, *n* = 13–16 fields. A minimum of 800 cells were quantified per condition.

### Cell viability assays

CellTiter-Glo 2.0 Luminescent assay (Promega Corporation) was used to determine cell viability for dose response assays. Cells at a density of 500 cells/well in white walled 384-well plates were drug treated 24 h following seeding. Following 48 h of treatment, CellTiter-Glo 2.0 was added to each well according to the manufacturer’s instructions. Luminescence was recorded using a PHERAstar FSX detection system (BMG Labtech). Following background value removal, data was normalised to untreated controls. Dose response curves were generated and drug potency values (IC_50_) calculated using the GraphPad Prism 8 software.

### Computational methods

#### Molecular modelling of CDCA3-Cdh1

A molecular model of CDCA3 was built based on the structure of *S. cerevisiae* APC/C activator protein Cdh1 bound to the three degrons of APC/C-CDH1 modulator 1 (PDB code: 4BH6)^52^. We report a molecular model of the WD40 propeller domain of human Cdh1 (Ser172-Ser474) and C-terminal domain of CDCA3 (Ser193-Glu267). The N-terminal domains outside of these sequences were not modelled, due to lack of homology with known 3D structures and the intrinsic disorder of the proteins. The KEN and D-box motifs of APC/C-Cdh1 modulator 1 were aligned with the two homologous degron sequences present in the C-terminal of CDCA3. We also looked for regions of negative charge in CDCA3 that could mimic the ABBA motif of APC/C-CDH1 modulator 1 and found a non-canonical ABBA-like motif from Arg220-Leu231. Firstly, the degrons motifs were modelled using position restraints and the remaining residues were then modelled from the template to CDCA3 using Modeller tool in UCSF Chimera. The *S. cerevisiae* cdh1 molecule is structurally homologous to human cdh1 (PDB code: 4UI9; chain R)^53^ and was therefore replaced using MatchMaker tool in UCSF Chimera^54^. The KEN, D box and non-canonical ABBA motifs of CDCA3 were modelled to interact with their respective sites on the Cdh1 molecule similar to the wild-type Cdk2-cyclinA2-Cks2-cdc20 complex (PDB code: 6Q6G)^55^.

#### Molecular dynamics simulations of CDCA3-cdh1 complex

Atomistic molecular dynamics (MD) simulation was applied to refine and explore the conformational space of the modelled CDCA3-cdh1 complex. The complex was solvated in explicit water using tleap from the AmberTools 16 package^56^. The Na+ ions were added to neutralise the system charge. The AMBER ff99SB*-ildn force field parameters^57^ and TIP3P model^58^ were used for the proteins/peptides and water molecules, respectively. MD simulation was performed using PMEMD.CUDA^59^ from AMBER 16 suite of programmes on NVIDIA GPU, which were compiled with CUDA 7.5. The electrostatic interactions were calculated using the Particle Mesh Ewald method with 12 Å short-range electrostatic and van der Waals cutoffs. All simulations were performed under periodic boundary conditions in a cubic water box. The equations of motion were solved using a time step of 2 fs. The SHAKE algorithm and Langevin dynamics were applied to constrain bonds to non-heavy atoms and to control the temperature. Each system was minimised using steepest descent for 1000 steps and conjugate gradient for another 500 steps. After minimisation, the system was heated from 0 to 300 K in 50 ps simulation by applying 10 kcal/(mol•Å^2^) harmonic position restraints to the protein and peptide heavy atoms with a constant particle number, volume and temperature (NVT) ensemble. Each system was further equilibrated in the constant particle number, pressure and temperature (NPT) ensemble at 1 atm and 300 K for 650 ps with same restraints as in the NVT run. Another 20 ns equilibration was performed by applying 2 kcal/(mol•Å^2^) harmonic position restraints on the KEN, D box and the non-cannonical ABBA motifs under NPT condition. Finally, 400 ns production run was performed to study the stability and the conformational changes in the peptide-protein complex. Snapshots from last 300 ns (30,000 frames) were combined for plotting secondary structures and obtaining stastistics on hydrogen bonds using the CPPTRAJ module of AmberTools 16. UCSF Chimera was used for visualisation of trajectory and preparing images of the molecular models.

### Bioinformatics analysis

Correlations between CDCA3 transcript expression and measures of genomic instability or response to chemotherapeutic drugs were assessed from The Cancer Genome Atlas (TCGA) RNAseq datasets. Specifically, log_2_-transformed level 3 Illumina HiSeq RNASeq V2 mRNA levels (RSEM) were used. Homologous recombination deficiency (HRD) scores were generated from a 230 multi-gene signature (Peng)^21^ or unweighted sum of three (loss of heterozygosity, telomeric allelic imbalances and large scale transitions) genomic scars (sum)^22^ as previously described. The phamacogenomic predictor of sensitivity to chemotherapy (PPSC) was determined according to a previously described multigene signature^23^. Somatic copy number variation analyses to assess chromosome arm gains or losses or the ploidy content of tumours were determined from SNP6 array copy number data as previously described^60^. Whole-genome doubling events were calculated using the ABSOLUTE method as previously described^61^. Within the two NSCLC histologies, the correlation between relative CDCA3 transcript expression and each parameter was assessed by linear regression analysis with *P*-values, *R*-values and 95% confidence intervals reported according to Spearman’s rank correlation. Differences in reported sample numbers between figures are due to availability of appropriate copy number or gene expression data. These analyses were performed in the *R* statistical environment (*R* Core Team, Vienna, Austria).

### Statistics and reproducibility

For in vitro experiments, data and statistical analyses were performed using GraphPad Prism V8 software. Results are shown as mean ± SD unless otherwise stated. Data were analysed using two-tailed Student’s *t* tests or Pearson correlation coefficients. *P*-values below 0.05 were considered significant and indicated using the following abbreviations: *P < 0.05, ****P < 0.0001.

### Reporting summary

Further information on research design is available in the Nature Research Reporting Summary linked to this article.

## Supplementary information

Supplementary Information

Description of Additional Supplementary Files

Supplemental Data 1

Reporting Summary

## Data Availability

Source data underlying figures is presented in Supplemental Data 1. Plasmids and other relevant data are available from the corresponding authors upon request.

## References

[1] Bray F (2018). Global cancer statistics 2018: GLOBOCAN estimates of incidence and mortality worldwide for 36 cancers in 185 countries. CA Cancer J. Clin..

[2] Siegel RL, Miller KD, Jemal A (2019). Cancer statistics, 2019. CA Cancer J. Clin..

[3] National Cancer Institute. *Cancer Stat Facts: Lung and Bronchus Cancer* https://seer.cancer.gov/statfacts/html/lungb.html (2019).

[4] Jamieson ER, Lippard SJ (1999). Structure, recognition, and processing of cisplatin− DNA adducts. Chem. Rev..

[5] Pilkington G (2015). A systematic review of the clinical effectiveness of first-line chemotherapy for adult patients with locally advanced or metastatic non-small cell lung cancer. Thorax.

[6] Stewart DJ (2007). Mechanisms of resistance to cisplatin and carboplatin. Crit. Rev. Oncol. Hematol..

[7] Gonzalez-Rajal A, Hastings J, Watkins DN, Croucher DR, Burgess A (2020). Breathing new life into the mechanisms of platinum resistance in lung adenocarcinoma. Front. Cell Dev. Biol..

[8] Ayad NG (2003). Tome-1, a trigger of mitotic entry, is degraded during G1 via the APC. Cell.

[9] Russell P, Nurse P (1987). Negative regulation of mitosis by wee1+, a gene encoding a protein kinase homolog. Cell.

[10] Uchida F (2012). Overexpression of cell cycle regulator CDCA3 promotes oral cancer progression by enhancing cell proliferation with prevention of G1 phase arrest. BMC Cancer.

[11] Hu Q (2015). OY-TES-1 may regulate the malignant behavior of liver cancer via NANOG, CD9, CCND2 and CDCA3: a bioinformatic analysis combine with RNAi and oligonucleotide microarray. Oncol. Rep..

[12] Yu J (2020). DNA hypomethylation promotes invasion and metastasis of gastric cancer cells by regulating the binding of SP1 to the CDCA3 promoter. J. Cell Biochem..

[13] Zhang Y (2019). CDCA3 is a potential prognostic marker that promotes cell proliferation in gastric cancer. Oncol. Rep..

[14] Qian W (2018). CDCA3 mediates p21-dependent proliferation by regulating E2F1 expression in colorectal cancer. Int. J. Oncol..

[15] Zhang W (2018). CDCA3 promotes cell proliferation by activating the NF-kappaB/cyclin D1 signaling pathway in colorectal cancer. Biochem Biophys. Res. Commun..

[16] Perez-Pena J (2017). Mitotic read-out genes confer poor outcome in luminal A breast cancer tumors. Oncotarget.

[17] Phan NN (2018). Distinct expression of CDCA3, CDCA5, and CDCA8 leads to shorter relapse free survival in breast cancer patient. Oncotarget.

[18] Adams MN (2017). Expression of CDCA3 is a prognostic biomarker and potential therapeutic target in non-small cell lung cancer. J. Thorac. Oncol..

[19] Kadouri L (2019). Homologous recombination in lung cancer, germline and somatic mutations, clinical and phenotype characterization. Lung Cancer.

[20] Telli ML (2016). Homologous Recombination Deficiency (HRD) Score predicts response to platinum-containing neoadjuvant chemotherapy in patients with triple-negative breast cancer. Clin. Cancer Res..

[21] Peng G (2014). Genome-wide transcriptome profiling of homologous recombination DNA repair. Nat. Commun..

[22] Marquard AM (2015). Pan-cancer analysis of genomic scar signatures associated with homologous recombination deficiency suggests novel indications for existing cancer drugs. Biomark. Res..

[23] Hess KR (2006). Pharmacogenomic predictor of sensitivity to preoperative chemotherapy with paclitaxel and fluorouracil, doxorubicin, and cyclophosphamide in breast cancer. J. Clin. Oncol..

[24] Shukla A (2020). Chromosome arm aneuploidies shape tumour evolution and drug response. Nat. Commun..

[25] Di Fiore B (2015). The ABBA motif binds APC/C activators and is shared by APC/C substrates and regulators. Dev. Cell.

[26] Ishida T, Kinoshita K (2007). PrDOS: prediction of disordered protein regions from amino acid sequence. Nucleic Acids Res..

[27] Buchan DWA, Jones DT (2019). The PSIPRED protein analysis workbench: 20 years on. Nucleic Acids Res..

[28] Davey NE, Morgan DO (2016). Building a regulatory network with short linear sequence motifs: lessons from the degrons of the anaphase-promoting complex. Mol. Cell.

[29] Zeng X (2010). Pharmacologic inhibition of the anaphase-promoting complex induces a spindle checkpoint-dependent mitotic arrest in the absence of spindle damage. Cancer Cell.

[30] Sharma K (2014). Ultradeep human phosphoproteome reveals a distinct regulatory nature of Tyr and Ser/Thr-based signaling. Cell Rep..

[31] Boeing S (2016). Multiomic analysis of the UV-induced DNA damage response. Cell Rep..

[32] Mertins P (2013). Integrated proteomic analysis of post-translational modifications by serial enrichment. Nat. Methods.

[33] Ferguson AD (2011). Structural basis of CX-4945 binding to human protein kinase CK2. FEBS Lett..

[34] Martins LR (2014). Activity of the clinical-stage CK2-specific inhibitor CX-4945 against chronic lymphocytic leukemia. Leukemia.

[35] Pierre F (2011). Discovery and SAR of 5-(3-chlorophenylamino)benzo[c][2,6]naphthyridine-8-carboxylic acid (CX-4945), the first clinical stage inhibitor of protein kinase CK2 for the treatment of cancer. J. Med. Chem..

[36] Siddiqui-Jain A (2010). CX-4945, an orally bioavailable selective inhibitor of protein kinase CK2, inhibits prosurvival and angiogenic signaling and exhibits antitumor efficacy. Cancer Res..

[37] van Gijn SE (2019). TPX2/Aurora kinase A signaling as a potential therapeutic target in genomically unstable cancer cells. Oncogene.

[38] Taube JM (2014). Association of PD-1, PD-1 ligands, and other features of the tumor immune microenvironment with response to anti-PD-1 therapy. Clin. Cancer Res..

[39] Mucaki EJ, Zhao JZL, Lizotte DJ, Rogan PK (2019). Predicting responses to platin chemotherapy agents with biochemically-inspired machine learning. Signal Transduct. Target Ther..

[40] Tsao MS, Yatabe Y (2019). Old soldiers never die: is there still a role for immunohistochemistry in the era of next-generation sequencing panel testing?. J. Thorac. Oncol..

[41] Nunez de Villavicencio-Diaz, T., Rabalski, A. J. & Litchfield, D. W. Protein kinase CK2: intricate relationships within regulatory cellular networks. *Pharmaceuticals*10.3390/ph10010027 (2017). 10.3390/ph10010027 PMC5374431 28273877

[42] Loizou JI (2004). The protein kinase CK2 facilitates repair of chromosomal DNA single-strand breaks. Cell.

[43] Barz T, Ackermann K, Dubois G, Eils R, Pyerin W (2003). Genome-wide expression screens indicate a global role for protein kinase CK2 in chromatin remodeling. J. Cell Sci..

[44] Luo H (2004). A new XRCC1-containing complex and its role in cellular survival of methyl methanesulfonate treatment. Mol. Cell Biol..

[45] von Morgen P (2017). MRE11 stability is regulated by CK2-dependent interaction with R2TP complex. Oncogene.

[46] Inoue H, Horiguchi M, Ono K, Kanoh J (2019). Casein kinase 2 regulates telomere protein complex formation through Rap1 phosphorylation. Nucleic Acids Res..

[47] Leimbacher PA (2019). MDC1 interacts with TOPBP1 to maintain chromosomal stability during mitosis. Mol. Cell.

[48] Meggio F, Pinna LA (2003). One-thousand-and-one substrates of protein kinase CK2?. FASEB J..

[49] Siddiqui-Jain A (2012). CK2 inhibitor CX-4945 suppresses DNA repair response triggered by DNA-targeted anticancer drugs and augments efficacy: mechanistic rationale for drug combination therapy. Mol. Cancer Ther..

[50] Zakharia K (2019). Preclinical in vitro and in vivo evidence of an antitumor effect of CX-4945, a casein kinase II inhibitor, in cholangiocarcinoma. Transl. Oncol..

[51] Pfleger CM, Lee E, Kirschner MW (2001). Substrate recognition by the Cdc20 and Cdh1 components of the anaphase-promoting complex. Genes Dev..

[52] He J (2013). Insights into degron recognition by APC/C coactivators from the structure of an Acm1-Cdh1 complex. Mol. Cell.

[53] Chang L, Zhang Z, Yang J, McLaughlin SH, Barford D (2015). Atomic structure of the APC/C and its mechanism of protein ubiquitination. Nature.

[54] Pettersen EF (2004). UCSF Chimera–a visualization system for exploratory research and analysis. J. Comput. Chem..

[55] Zhang S, Tischer T, Barford D (2019). Cyclin A2 degradation during the spindle assembly checkpoint requires multiple binding modes to the APC/C. Nat. Commun..

[56] Case, D. A. et al. AMBER 2016. University of California, San Francisco (2016).

[57] Lindorff-Larsen K (2010). Improved side-chain torsion potentials for the Amber ff99SB protein force field. Proteins.

[58] Jorgensen, W. I. *Am. Chem. Sot*. 106 (1984) 6638. WL Jorgensen, J. Chandrasekhar, JD Madura, RW Impey and ML Klein. *J. Chem. Phys* **79**, 0 (1983).

[59] Salomon-Ferrer R, Götz AW, Poole D, Le Grand S, Walker RC (2013). Routine microsecond molecular dynamics simulations with AMBER on GPUs. 2. Explicit solvent particle mesh Ewald. J. Chem. Theory Comput..

[60] Thangavelu, P. U. et al. Overexpression of the E2F target gene CENPI promotes chromosome instability and predicts poor prognosis in estrogen receptor-positive breast cancer. *Oncotarget* **8**, 62167–62182 (2017). 10.18632/oncotarget.19131 PMC5617495 28977935

[61] Carter SL (2012). Absolute quantification of somatic DNA alterations in human cancer. Nat. Biotechnol..

