# Supplementary material for: Elevating CDCA3 levels in non-small cell lung cancer enhances sensitivity to platinum-based chemotherapy
Source: Commun Biol. 2021 May 28;4:638. doi: 10.1038/s42003-021-02136-8 (PMC8163776; doi:10.1038/s42003-021-02136-8)

Figure legends

Supplementary Fig. 1:

a,b, Scatter plots showing linear regression analysis of the correlation between relative *CDCA3* mRNA levels and the number of chromosome arm gains and losses in ADC (**a**) and SqCC cancers (**b**). *R* and *P* values determined according to Spearman's rank correlation. **c,d,** Violin plots showing relative *CDCA3* mRNA levels in diploid and aneuploid ADC (**c**) and SqCC cancers (**d**). e,**f,** Violin plots showing relative *CDCA3* mRNA levels in ADC (**e**) and SqCC cancers (**f**) exhibiting no whole-genome doublings, a single whole-genome duplication or two whole-genome doublings. **c-f,** *P* values as shown determined using unpaired Student's *t* test. **g,** *Left,* dose response curves for eight NSCLC cell lines treated with escalating doses of cisplatin. *Middle*, dose response curves for seven NSCLC cell lines treated with escalating doses of carboplatin. Cells were treated with cisplatin or carboplatin for 48 hours before assessing cell viability. *Right panel*, Cisplatin and carboplatin potency values (IC_{50}) were calculated using GraphPad Prism and listed for each cell line. n = 4 (cisplatin), n = 3 (carboplatin). **h,** Cells endogenously expressing high versus low CDCA3 levels were untreated, cisplatin treated for 12 h (cisplatin) or cultured in fresh growth medium for 8 h following cisplatin treatment (recovery). Data points represent an average of γH2AX foci/nuclei per field of view from a minimum of 800 nuclei (n = 13-16 fields). Blue lines indicate median values. Dotted black lines highlight change in foci count following recovery. Percent recovery calculated by difference between 100% and ratio of recovery/cisplatin expressed as a percentage. **i,** Representative CellProfiler software images indicating detection of nuceli (*left panel*), foci (*middle panel*) and merged foci within detected nuclei (*right panel*).

Supplementary Fig. 2:

a,b, Amino acid sequence analysis of the full length CDCA3 to predict the presence of disordered regions (PrDOS, **a**) and secondary structures (PSIPRED, **b**). **a,** Ordered and disordered predictions are overlayed the full length CDCA3 sequence with legend describing predictions listed within figure (*lower right*). **b,** Secondary structure predictions indicated above each amino acid of the full length CDCA3 sequence. Legend describing predicted secondary structures listed in figure (*lower right*). C, coil; H, helix.

Supplementary Fig. 3:

a, CDCA3 protein levels determined by immunofluorescence and high throughput microscopy of cells transfected with control siRNA or 62 siRNAs targeting expression of 38 different serine/threonine protein kinases. Microscopy was performed 36 hours following transfection. Protein levels were determined using CellProfiler software analysis and reported as normalised to siRNA control cells. A minimum of 1000 cells were analysed. **b,** Endogenous CDCA3 western blot analysis of eight NSCLC cell lines treated with or without CX-4945 for 12 hours. Phosphorylated CK2 substrate probe to assess impact of CK2 inhibition with CX-4945. Tubulin was used as a loading control.

Supplementary Fig. 4:

a, Western blot analysis of lysates from CRL-5889, EBC-1 and HTB-182 cells transfected with empty vector or CDCA3-FLAG. Transfected cells as used in FLAG and CDCA3 western blot analysis was performed to detect ectopic and endogenous CDCA3 respectively. Tubulin was used as a loading control. *Arrowhead*, ectopic CDCA3. *Asterix*, endogenous CDCA3. Representative western blot analysis from three independent experiments. Corresponds with Fig. 6a,b. **b,** Dose response curves for eight NSCLC cell lines treated with escalating doses of CX-4945. Cells were treated with CX-4945 for 48 hours before assessing cell viability. *Right panel*, CX-4945 potency values (IC_{50}) were calculated using GraphPad Prism and listed for each cell line. n = 4.

Supplemental Figure 1

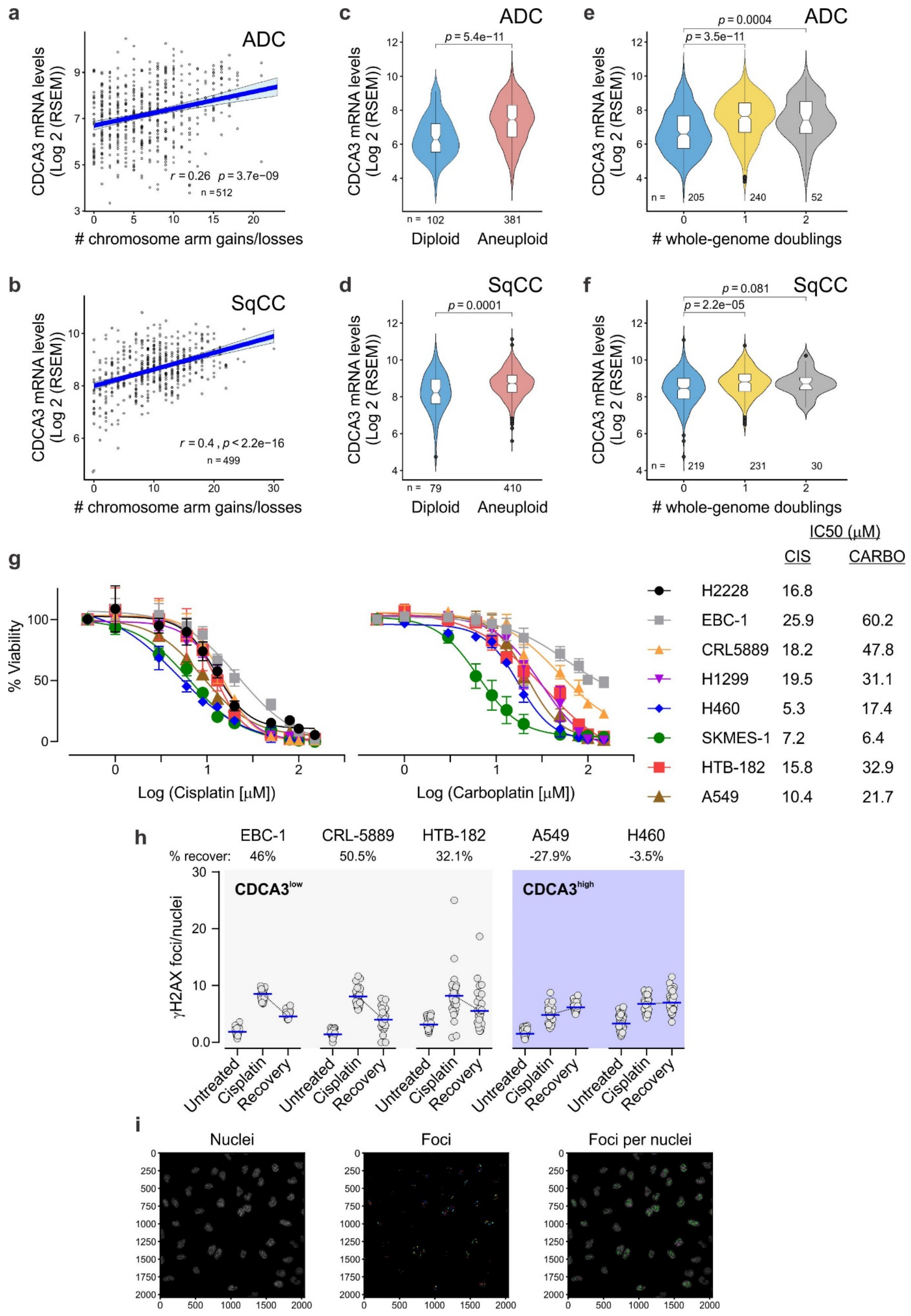

Supplemental Figure 2

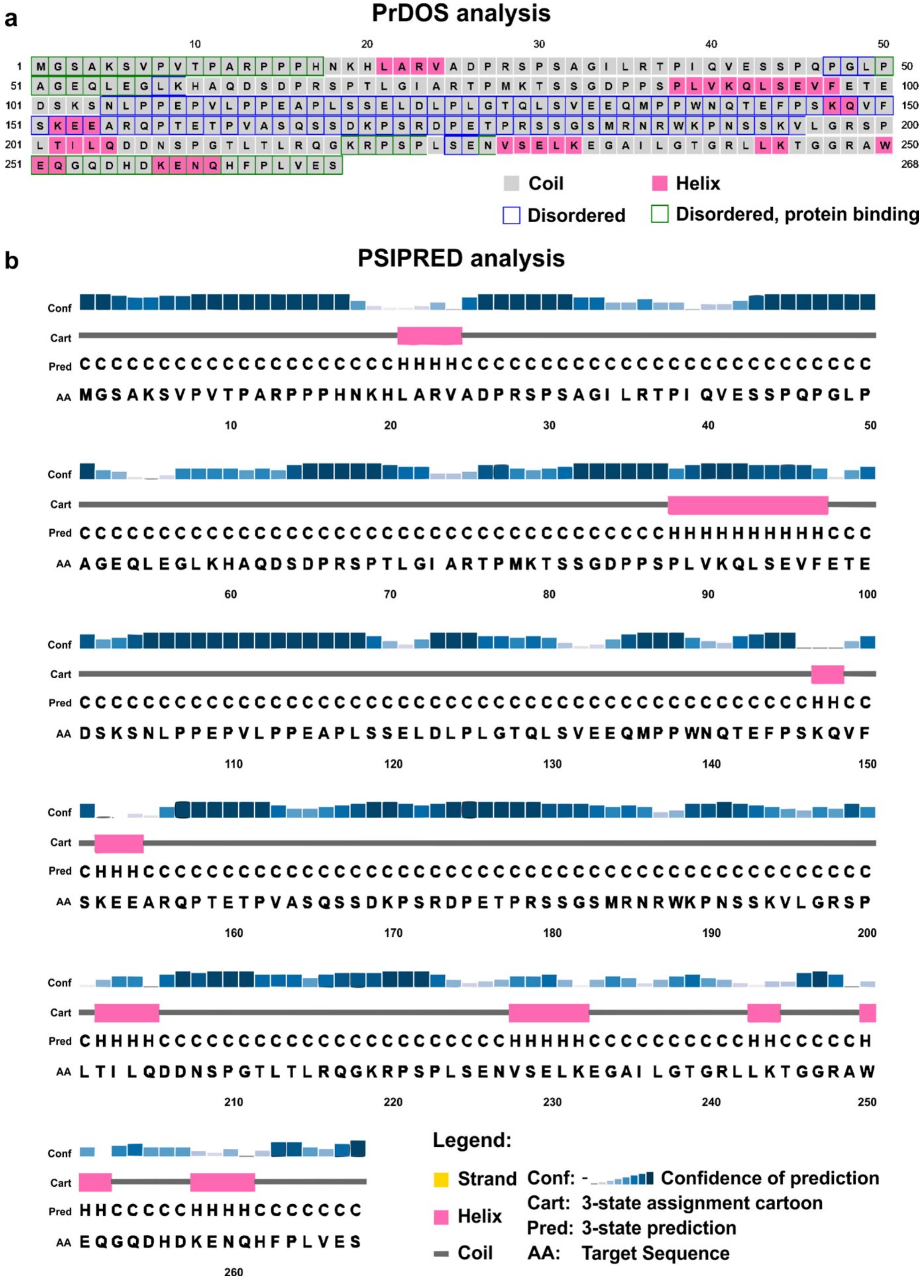

Supplemental Figure 3

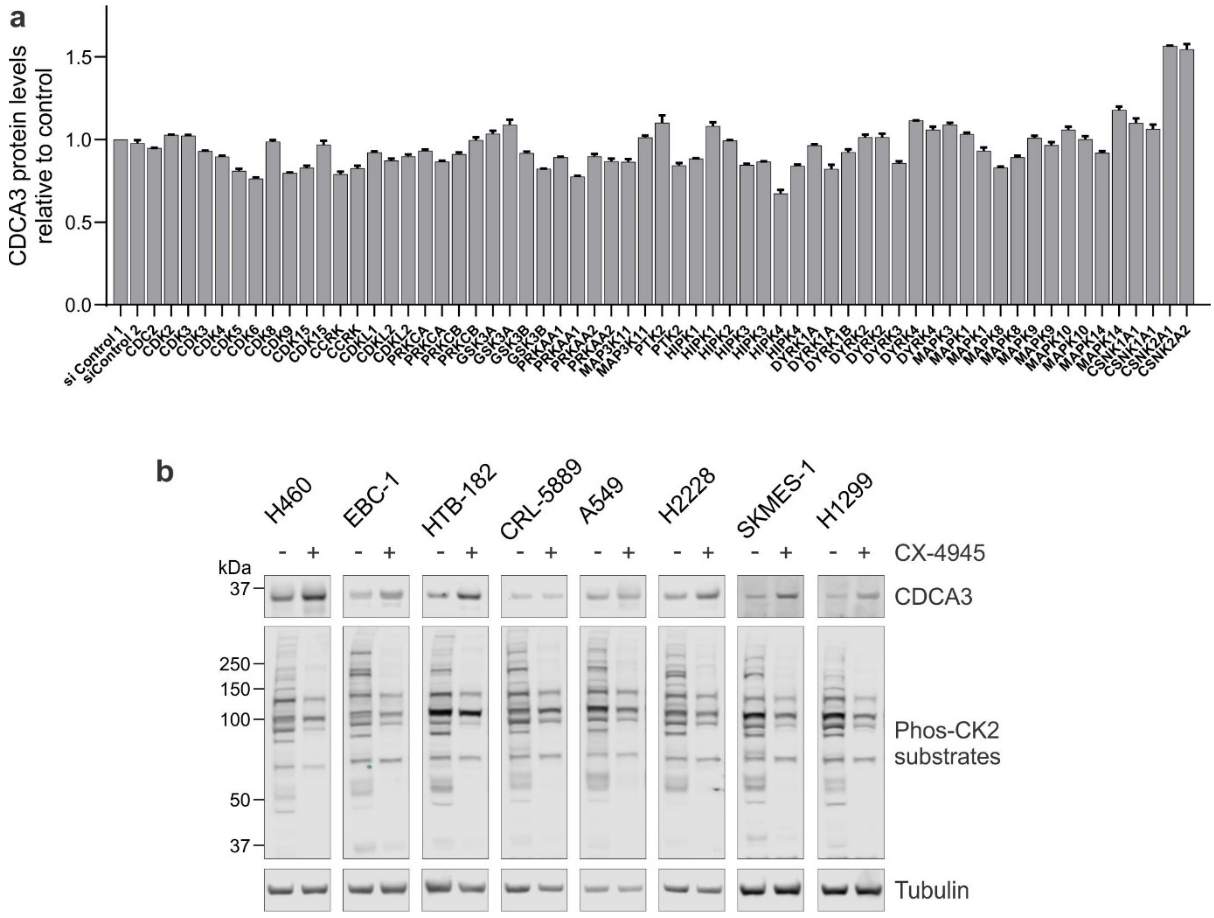

Supplemental Figure 4

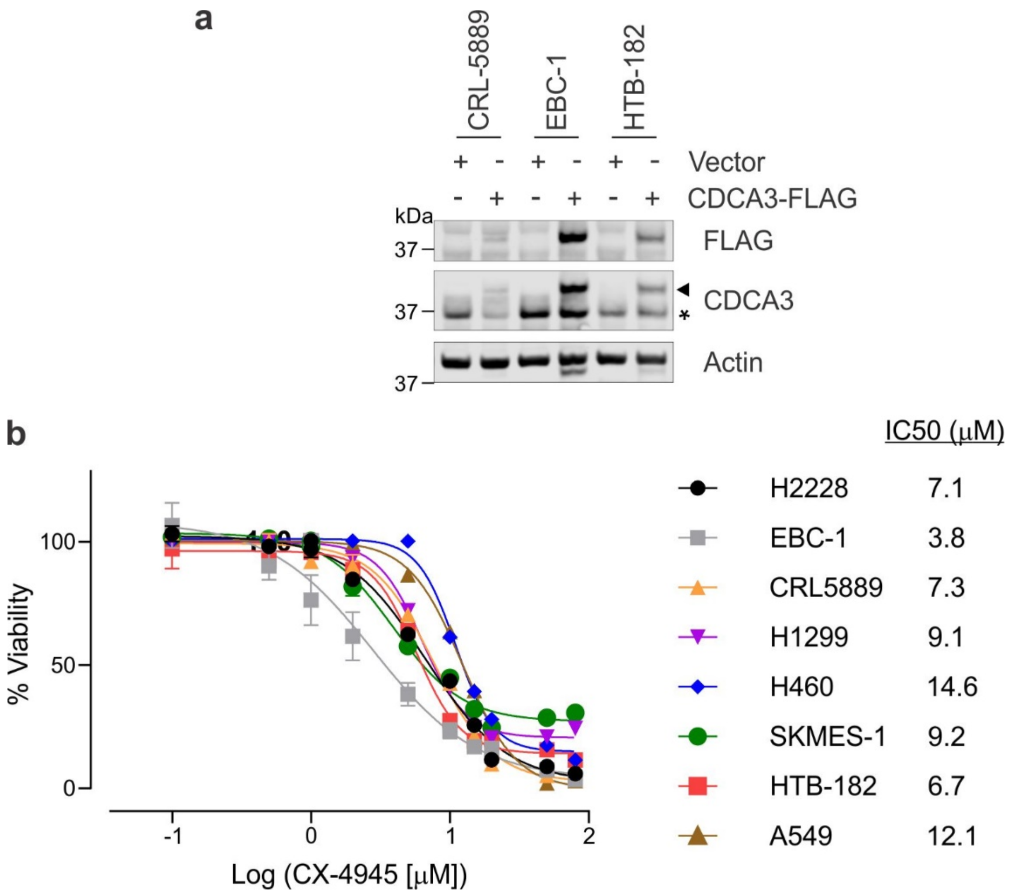

Supplement: Supplementary file 1 — Supplementary Information [file 42003_2021_2136_MOESM1_ESM.pdf]
