# Supplementary material for: Elevating CDCA3 levels in non-small cell lung cancer enhances sensitivity to platinum-based chemotherapy
Source: Commun Biol. 2021 May 28;4:638. doi: 10.1038/s42003-021-02136-8 (PMC8163776; doi:10.1038/s42003-021-02136-8)
Supplement: Supplementary file 2 — Description of Additional Supplementary Files [file 42003_2021_2136_MOESM2_ESM.pdf]

Description of Additional Supplementary Files

File name: Supplemental Data 1
Description: Source data underlying figures is presented in Supplemental Data 1.
