# Supplementary material for: Elevating CDCA3 levels in non-small cell lung cancer enhances sensitivity to platinum-based chemotherapy
Source: Commun Biol. 2021 May 28;4:638. doi: 10.1038/s42003-021-02136-8 (PMC8163776; doi:10.1038/s42003-021-02136-8)
Supplement: Supplementary file 4 — Reporting Summary [file 42003_2021_2136_MOESM4_ESM.pdf]

nature research

Corresponding author(s): Mark N. Adams

Last updated by author(s): Jan 6, 2021

Reporting Summary

Nature Research wishes to improve the reproducibility of the work that we publish. This form provides structure for consistency and transparency in reporting. For further information on Nature Research policies, see our Editorial Policies and the Editorial Policy Checklist.

Statistics

For all statistical analyses, confirm that the following items are present in the figure legend, table legend, main text, or Methods section.

n/a	Confirmed	
☐	☒	The exact sample size (*n*) for each experimental group/condition, given as a discrete number and unit of measurement
☐	☒	A statement on whether measurements were taken from distinct samples or whether the same sample was measured repeatedly
☐	☒	The statistical test(s) used AND whether they are one- or two-sided<br>*Only common tests should be described solely by name; describe more complex techniques in the Methods section.*
☒	☐	A description of all covariates tested
☐	☒	A description of any assumptions or corrections, such as tests of normality and adjustment for multiple comparisons
☐	☒	A full description of the statistical parameters including central tendency (e.g. means) or other basic estimates (e.g. regression coefficient) AND variation (e.g. standard deviation) or associated estimates of uncertainty (e.g. confidence intervals)
☐	☒	For null hypothesis testing, the test statistic (e.g. *F*, *t*, *r*) with confidence intervals, effect sizes, degrees of freedom and *P* value noted<br>*Give P values as exact values whenever suitable.*
☒	☐	For Bayesian analysis, information on the choice of priors and Markov chain Monte Carlo settings
☒	☐	For hierarchical and complex designs, identification of the appropriate level for tests and full reporting of outcomes
☐	☒	Estimates of effect sizes (e.g. Cohen's *d*, Pearson's *r*), indicating how they were calculated

Our web collection on statistics for biologists contains articles on many of the points above.

Software and code

Policy information about availability of computer code

Data collection	No software was used.
Data analysis	Software: AmberTools 16 package (computational modelling); R statistical environment (bioinformatics analysis); GraphPad Prism V8 (statistical analysis)

For manuscripts utilizing custom algorithms or software that are central to the research but not yet described in published literature, software must be made available to editors and reviewers. We strongly encourage code deposition in a community repository (e.g. GitHub). See the Nature Research guidelines for submitting code & software for further information.

Data

Policy information about availability of data

All manuscripts must include a data availability statement. This statement should provide the following information, where applicable:

- Accession codes, unique identifiers, or web links for publicly available datasets
- A list of figures that have associated raw data
- A description of any restrictions on data availability

All protein structures used in our computational methods are available from the Protein Data Bank (PBD codes 4BH6 and 4UI9). Previously generated RNAseq datasets are available from The Cancer Genome Atlas. Raw data for graphs and uncropped western blot images are available as supplemental data. (Uncropped images are being prepared during manuscript review).

Field-specific reporting

Please select the one below that is the best fit for your research. If you are not sure, read the appropriate sections before making your selection.

- [x] Life sciences
- [ ] Behavioural & social sciences
- [ ] Ecological, evolutionary & environmental sciences

For a reference copy of the document with all sections, see nature.com/documents/nr-reporting-summary-flat.pdf

Life sciences study design

All studies must disclose on these points even when the disclosure is negative.

Sample size	No sample size calculation was performed. For in vitro experiments, a minimum of three independent experiments were performed.
Data exclusions	No data were excluded from the analyses.
Replication	Experiments were performed a minimum of three times. For viability assays, these were performed on three independent occasions in triplicate. All replicates are included within the manuscript.
Randomization	Samples were blindly randomised for inclusion within experiments. For example, following of seeding cells into dishes for in vitro experiments, dishes were selected at random for either drug treatment (verus vehicle) or for transfection.
Blinding	Authors were not blinded during in vitro data collection.

Reporting for specific materials, systems and methods

We require information from authors about some types of materials, experimental systems and methods used in many studies. Here, indicate whether each material, system or method listed is relevant to your study. If you are not sure if a list item applies to your research, read the appropriate section before selecting a response.

Materials & experimental systems

n/a	Involved in the study	
☐	☒	Antibodies
☐	☒	Eukaryotic cell lines
☒	☐	Palaeontology and archaeology
☒	☐	Animals and other organisms
☒	☐	Human research participants
☒	☐	Clinical data
☒	☐	Dual use research of concern

Methods

n/a	Involved in the study	
☒	☐	ChIP-seq
☒	☐	Flow cytometry
☒	☐	MRI-based neuroimaging

Antibodies

Antibodies used	The CDCA3 antibody (HPA026587), monoclonal FLAG M2 antibody (F1804) and α-Tubulin antibody (T9026) were purchased from Sigma Aldrich. Antibodies against the HA tag (#3724) and phospho-CK2 substrate motif (#8738) were purchased from Cell Signaling Technology (Genesearch, Australia). The Cdh1 (ab77885) and γH2AX (ab26350) antibodies were from Abcam. The FANCI antibody (A301-254A) was from Bethyl Laboratories. Donkey anti-rabbit and anti-mouse Alexa Fluor 488 antibodies were purchased from Life Technologies.
Validation	Antibody specificity was validated in-house using siRNA or pharmacological techniques.

Eukaryotic cell lines

Policy information about cell lines

Cell line source(s)	All cell lines are commercially available. All cells with the were from the American Type Culture Collection (ATCC) with the exception of EBC-1 which were purchased from Cell Bank Australia.
Authentication	Frozen stocks of all cell lines were made within 5 passages of purchase from vendors. All experiments were performed on cells grown from original stocks. To verify technique, several cell lines were STR profiled at the Genomics Research Centre (Brisbane, Australia).
Mycoplasma contamination	All cell lines tested negative for mycoplasma.
Commonly misidentified lines (See ICLAC register)	*Name any commonly misidentified cell lines used in the study and provide a rationale for their use.*
